# Targeting treatment resistance: unveiling the potential of RNA methylation regulators and TG-101,209 in pan-cancer neoadjuvant therapy

**DOI:** 10.1186/s13046-024-03111-x

**Published:** 2024-08-19

**Authors:** Yaoyao Zhou, Ziyun Liu, Cheng Gong, Jie Zhang, Jing Zhao, Xia Zhang, Xiangyu Liu, Bin Li, Rui Li, Zhenyu Shi, Yongjie Xie, Li Bao

**Affiliations:** 1 Tianjin Medical University Cancer Institute & Hospital, National Clinical Research Center for Cancer, Tianjin, China; 2 Tianjin Medical University, Ministry of Education, Tianjin, China; 3 Tianjin’s Clinical Research Center for Cancer, Tianjin, China; 4 Key Laboratory of Breast Cancer Prevention and Therapy, Tianjin Medical University, Ministry of Education, Tianjin, China; 5 Key Laboratory of Cancer Prevention and Therapy, Tianjin, China; 6 Liver Cancer Center, Tianjin Medical University Cancer Institute and Hospital, Tianjin, China; 7 Department of Gynecological Oncology, Tianjin Medical University Cancer Institute and Hospital, National Clinical Research Center for Cancer, Tianjin, China; 8 Department of Gastric Surgery, Key Laboratory of Digestive Cancer, Tianjin, China; 9 Department of Clinical Laboratory, Tianjin Medical University Cancer Institute and Hospital, Tianjin, China; 10 Department of Breast Oncoplastic Surgery, Tianjin Medical University Cancer Institute, Tianjin, 300060 China; 11 State Key Laboratory of Druggability Evaluation and Systematic Translational Medicine, Tianjin, China

**Keywords:** RNA methylation regulators, Chemotherapy, Immunotherapy, Pan-cancer, TG-101209

## Abstract

**Background:**

Tumor recurrence and mortality rates remain challenging in cancer patients despite comprehensive treatment. Neoadjuvant chemotherapy and immunotherapy aim to eliminate residual tumor cells, reducing the risk of recurrence. However, drug resistance during neoadjuvant therapy is a significant hurdle. Recent studies suggest a correlation between RNA methylation regulators (RMRs) and response to neoadjuvant therapy.

**Methods:**

Using a multi-center approach, we integrated advanced techniques such as single-cell transcriptomics, whole-genome sequencing, RNA sequencing, proteomics, machine learning, and in vivo/in vitro experiments. Analyzing pan-cancer cohorts, the association between neoadjuvant chemotherapy/immunotherapy effectiveness and RNA methylation using single-cell sequencing was investigated. Multi-omics analysis and machine learning algorithms identified genomic variations, transcriptional dysregulation, and prognostic relevance of RMRs, revealing distinct molecular subtypes guiding pan-cancer neoadjuvant therapy stratification.

**Results:**

Our analysis unveiled a strong link between neoadjuvant therapy efficacy and RNA methylation dynamics, supported by pan-cancer single-cell sequencing data. Integration of omics data and machine learning algorithms identified RMR genomic variations, transcriptional dysregulation, and prognostic implications in pan-cancer. High-RMR-expressing tumors displayed increased genomic alterations, an immunosuppressive microenvironment, poorer prognosis, and resistance to neoadjuvant therapy. Molecular investigations and in vivo/in vitro experiments have substantiated that the JAK inhibitor TG-101,209 exerts notable effects on the immune microenvironment of tumors, rendering high-RMR-expressing pan-cancer tumors, particularly in pancreatic cancer, more susceptible to chemotherapy and immunotherapy.

**Conclusions:**

This study emphasizes the pivotal role of RMRs in pan-cancer neoadjuvant therapy, serving as predictive biomarkers for monitoring the tumor microenvironment, patient prognosis, and therapeutic response. Distinct molecular subtypes of RMRs aid individualized stratification in neoadjuvant therapy. Combining TG-101,209 adjuvant therapy presents a promising strategy to enhance the sensitivity of high-RMR-expressing tumors to chemotherapy and immunotherapy. However, further validation studies are necessary to fully understand the clinical utility of RNA methylation regulators and their impact on patient outcomes.

**Supplementary Information:**

The online version contains supplementary material available at 10.1186/s13046-024-03111-x.

## Introduction

Cancer is not be underestimated public health problem in the world, which has become the second most lethal disease in the United States after heart disease [1]. Medication is a selective available to improve clinical benefits for cancer patients, including chemotherapy, neoadjuvant chemotherapy, targeted therapy, endocrine therapy, immunotherapy, and other therapies. Preoperative neoadjuvant chemotherapy was administered before curative-intent surgery in treatment-naïve patients to reduce the size of the tumor and improve the surgical resectability [2]. Neoadjuvant therapy followed by surgery could improve patient survival and reduce tumor recurrence [3]. However, multiple physiological and pathological factors can lead to cancer resistance [4]. The therapeutic effect of drugs on tumors was limited and there was no accurate molecular phenotype to guide tumor treatment.

RNA methylation modifications, mainly including m6A, m5C, m1A, and m7G, have been widely reported in promoting tumor progression and development. The enzymes regulating the methylation levels of RNAs can be categorized as “writers,” “erasers,” and “readers” according to their function. Writers install methylation on RNA, while erasers tend to remove modifications from RNAs. Readers can identify methylation on RNAs and carrier-conserved structures recognized as functional domains by the corresponding proteins. Among these RNA methylation modifications, m6A RNA methylation is regarded as the most prevalent and abundant RNA modification in eukaryotic cells, influencing RNA stability and translational speed [5, 6]. m6A sites are produced by adding a methyl group to the sixth nitrogen atom position of adenosine and are often generated at the consensus motif of RRACH (R represents G or A, and H represents C, A or U) and DRACH (D represents A, G or U) [7, 8]. m6A methylation plays a vital role in mammals, and they are involved in processes including stress responses, neurogenesis, embryonic development, sex determination, circadian rhythm, and tumorigenesis [9, 10]. It has been demonstrated that genetic and transcriptional changes in m6A enzymes influence cell proliferation, apoptosis, developmental defects, impaired self-renewal capacity, tumor progression and metastasis, and immune dysregulation [11–13]. m5Cs in RNAs mainly include NOL1/NOP2/SUN domain (NSUN) family members containing seven proteins (NSUN1-7) in humans and the DNA methyltransferase (DNMT) homolog DNMT2 [14]. In all members of the NSUN family, the catalytic cysteine is preceded by threonine, while the DNMT family uses conserved glutamate to promote covalent bond formation [15]. m5Cs are involved in multiple processes, including mRNA export [16], RNA stability [17], long-distance transport [18], translation, alternative splicing of viral RNA [19], stress response [20], and tumor progression and migration [21]. m1A refers to methylation at the N1 position of RNA base A and occurs in tRNA, rRNA, mRNA, and mitochondrial (mt) transcripts [22]. It was first found to be a highly conserved modification in tRNA and rRNA. Then, it was also reported to be present in mRNA via m1A-seq and other mapping technologies [23]. m1A occurs in 0.015–0.054% of total cytosine residues in mammalian mRNA [24], and the relatively low incidence may be due to the difficulty in mapping m1A on the transcriptome. In most cancer subtypes, the m1A demethylase ALKBH3 plays an oncogenic role in the development of cancer through various regulatory mechanisms [25]. The TRM6–TRM61 complex is an m1A writer that catalyses methylation on rRNA, tRNA, and mRNA, but this only occurs with a small probability [26]. N7-methylguanosine (m7G), one of the conserved modifications in eukaryotic RNA, usually appears at the 5’ caps of mRNA or internal positions in rRNA and tRNA of various species [27]. It has been documented that m7G modification participates in several disorders, including chromosomal abnormality diseases, excessive stem cell proliferation, and aberrant differentiation [28, 29]. m7G methylation also plays a crucial role in tumor development and is correlated with diverse biological activities in tumorigenesis [30]. Among m7G regulators, METTL1 methyltransferase is well characterized and participates in various tumor-related processes [31]. Its dysregulation is related to cell cycle progression, tumor invasion, cancer cell migration, and tumor metastasis.

It had been reported that RNA methylation regulators were related to treatment resistance in cancer. Many studies have demonstrated that m6A was associated with cisplatin resistance in tumors. Cancer cell sensitivity to cisplatin was recovered by ALKBH5 knockdown or inhibition of the JAK/STAT3 signaling pathway [32]. In pancreatic cancer, METTL3 modulates the MAPK pathway to enhance the resistance of pancreatic tumors to chemotherapy and radiotherapy [33]. In the HeLa cell line, the combined knockdown of NSUN2 and METTL1 increased the sensitivity of cells to 5-FU [34]. YTHDF1 depletion restores CD8 + T cell activity to inhibit cancer growth and promotes cancer cell death in combination with PD1 inhibitors [35]. Elimination of the m6A methylation transferase METTL3/METTL14 increases the efficacy of anti-PD1 in colorectal cancer and melanoma patients [36]. However, there is no precise molecular typing of RNA methylation to guide pan-cancer treatment. Hence, exploring how RNA methylation affects the mechanism of tumor drug resistance and constructing RMRs stratification therapy become a key focus for us.

By comparing single-cell transcriptome levels of treatment non-responders and responders in pan-cancer, RNA methylation was proven to correlate with cancer treatment resistance. For the first time, we combined multi-center single-cell sequencing, whole genome sequencing, transcriptome sequencing, proteomic data, and *in vivo/in vitro* experiments, which depicted RMRs’ widespread genomic variation, transcriptomic dysregulation, proteomic abnormalities, and poor patient prognosis in pan-cancer. Random forest machine learning algorithm and multi-omic data established accurate RMRs subtypes guiding cancer therapy strategies. The high-expression RMRs group was more malignant and insensitive to traditional chemotherapy drugs or immune checkpoint inhibitors across cancer types. Based on small molecule drug prediction, RNA sequencing of our cohort, and *in vivo/in vitro* pan-cancer cell line experiments, we predicted and validated the effective JAK inhibitor TG-101,209. The co-administration of TG-101,209 increased effector CD8 + T cells and enhanced sensitivity to neoadjuvant chemoimmunotherapy in high-RMR-expressing tumors. This study illustrated that RMRs took an important role in pan-cancer progression, tumor environment components, patients’ prognosis, and therapy. RMRs’ molecular subtype provided precise stratification guidance for neoadjuvant therapy. A combination of TG-101,209 and neoadjuvant chemoimmunotherapy will be an effective treatment strategy for high-RMR-expressing tumors in pan-cancer.

## Methods

### Patient and public datasets collection

The study protocol complianced with the ethical guidelines and was accepted by the Clinical Research Ethics Committee of Tianjin Medical University Cancer Hospital. 16 pancreatic cancer specimens were obtained from the Tianjin Medical University Cancer Hospital and were performed RNA sequencing. All the patients provided written informed consent. Single-cell RNA datasets and bulk transcriptomes of patients who received chemotherapy or immunotherapy were collected from the GEO database under a specific GEO accession number. The normalized mRNA expression data (FPKM data), somatic mutation, copy number variation (CNV), and clinical data of 33 cancer types were downloaded from the TCGA database (https://portal.gdc.cancer.gov/). The CNV segmentation data were processed by the GISTIC 2.0 algorithm (https://www.broadinstitute.org/cancer/cga/gistic) to identify the amplification and deletion events. The somatic mutation frequency of RMRs was calculated for samples with nonsilent mutations. Single nucleotide variations (SNV) data was processed by the “maftools” package. The cell line expression data were obtained from the (Genomics of Drug Sensitivity in Cancer) GDSC database (https://www.cancerrxgene.org/), and the IC50 value matrix was also included for drug sensitivity analysis. The Human Protein Atlas (https://www.proteinatlas.org/) database was adopted to collect immunohistochemistry images of tumors and normal tissues.

### RNA-sequencing library construction

Strand-specific libraries were constructed using the TruSeq Stranded mRNA Sample Prep Kit (Illumina) according to the manufacturer’s instructions. Poly-adenylated RNA from intact total RNA was refined using oligo-dT beads. The complementary DNA fragment was 3′ end adenylated and ligated to Illumina paired-end sequencing adapters and amplified by PCR. The libraries were sequenced with 65 base pair (bp) single-end reads on a HiSeq 2500 System in high output mode using V4 chemistry (Illumina). Raw reads were aligned to GRCh38 using a STAR RNA-seq aligner after which gene expression levels were quantified by Salmon (see URLs) using default parameters for both applications.

### Single-cell RNA sequencing data analysis

We used the “Seurat” R package to eliminate low-quality cells, normalize scRNA-seq data, and cluster different cell types. The reference data from the Human Primary Cell Atlas and representative marker genes annotated different single-cell clusters. We used the Wilcoxon rank sum test and “FindMarkers” Seurat functions for differential gene analysis. Significant genes were determined with *P* < 0.05. The “CellChat” R package was employed to construct communication networks between cell subpopulations [37].

### Unsupervised clustering of 46 RMRs

Before clustering, we normalized the RMR expression in each cancer type by using the sweep function to subtract the median expression value for each RMR. Then, we combined RMR expression data from all cancers, used the “ConsensusClusterPlus” R package for unsupervised clustering, chose the KM clustering algorithm, and conducted 1000 repetitions to ensure stability.

### Random forest (RF) model construction and validation

The feature matrix was 46 RMRs median centered expression data of the TCGA samples, and the samples belonged to three clusters as an outcome variable. The R package caret was used to train the model through a random forest algorithm, and the training algorithm was optimized via the following two important parameters: tenfold cross-validation resampling method and parameter range less than the number of features. The predict function was implemented in the R package randomForest. We performed tenfold cross-validation, and the area of the ROC curve was calculated and plotted by the “survivalROC” package.

### Gene set variation analysis (GSVA), Gene set enrichment analysis (GSEA), Kyoto encyclopedia of genes and genomes (KEGG), and Gene ontology (GO) annotation

To study the differences in the RMRs clusters in biological processes, we used the “GSVA” R package to conduct GSVA enrichment analysis. Single-gene GSEA was used to annotate the RMRs-related pathways, and the pathways with enrichment *P* values < 0.05 were considered significant. KEGG and GO analysis for RMR-related genes was performed in the R package ‘clusterProfiler’ with a cut-off value of *P* < 0.05. The gene sets “hallmark.v7.4” and “c2.cp.kegg.v7.4” for pathway enrichment analysis were downloaded from the MSigDB database.

### Calculation of TME (Tumor Microenvironment) cell infiltration in RMRs clusters

We used the xCell algorithm to examine whole-tumor gene expression data to score the relative abundance across tumors of 64 types of immune and stromal cells. The ssGSEA algorithm was employed to quantify the relative abundance of 28 types of immune cells in multiple cancer types. The comparison of immune cell infiltration among the three clusters used the Kruskal‒Wallis test with a *P-*value threshold of 0.05.

### Prediction of potential targeted drugs against RMRs

The Connectivity map analysis (CMap) online tool (http://clue.io) has been demonstrated to be useful in silico drug screening tools to target disorders. This tool was formally developed and widely used in 2007 to discover the mechanisms of small-molecule drugs [38]. It was further refined in 2017, complementing the L1000 platform to more accurately identify small-molecule drugs [39]. The CMap helped to unearth targeted drugs for SPOCK1 in lung cancer [40]. Multi-omics analysis and CMap association to identify drug candidates that may restore ATRX-deficient transcriptional changes in gliomas [41]. The top 20 genes that positively correlated with RMRs were subjected, and the query result was a list of drugs with a “connectivity score” ranging from + 1 (positive connectivity) to -1 (negative connectivity). Drugs with a positive connectivity score may generate similar gene expression outcomes with the state of interest (query state), whereas those with a negative score produce reverse gene expression patterns with the query. Drugs with consistently negative scores in most cancers were potential drugs targeting RMRs, we tended to choose JAK inhibitor because it appeared the most frequently.

### Reverse transcription PCR (RT-PCR)

According to the manufacturer’s instructions, the total RNA was extracted from tumor tissues and adjacent normal tissues using TRIzol (Invitrogen). The cancer types were Pancreatic adenocarcinoma (PAAD), bladder cancer (BLCA), Breast invasive carcinoma (BRCA), Colon adenocarcinoma (COAD), Rectum adenocarcinoma (READ), Liver hepatocellular carcinoma (LIHC), Lung squamous cell carcinoma (LUSC), Esophageal carcinoma (ESCA), Stomach adenocarcinoma (STAD), and Ovarian cancer (OV). For each cancer type, we selected 6 pairs of tissue samples. Then, the mRNA was used for first-strand cDNA synthesis with the Reverse Transcription PCR System (Bimake), and the cDNA levels were analyzed by real-time fluorescence quantitative PCR. Each RT-PCR experiment was independently repeated at least three times. β-actin was used as a positive control. The relevant primers were listed in the supplementary table (Table S3).

### Western blot

Cells were lysed using RIPA lysis mixed with phosphatase inhibitors and protease inhibitors. Protein concentrations were measured by BCA protein assay. The same amount of protein was separated by sodium dodecyl sulfate-polyacrylamide gel electrophoresis (SDS-PAGE). Membranes with blot proteins were incubated overnight at 4 °C with related antibodies which were diluted at 1:1000. Horseradish peroxidase-conjugated goat anti-rabbit antibody and goat anti-mouse antibody as secondary antibody were diluted at 1:5000. The blots were detected with a Chemi-Scope exposure machine. Antibodies for western blot are supplemented in Table S13.

### Cell culture

The human breast cancer cell line MCF-7, human liver cancer cell line Hep-G2, human lung cancer cell line A-549, human colorectal cancer cell line SW480, and human pancreatic cancer cell line SW1990 were obtained from Tianjin Cancer Institute. The murine breast cancer cell line 4T1 and murine lung cancer cell line LLC1 were obtained from the Type Culture Collection Committee of the Chinese Academy of Sciences (Shanghai, China). The murine colorectal cancer cell line MC38 was purchased from Korean Cell Line Bank (Seoul, Republic of Korea). The murine hepatocellular carcinoma cell line Hepa1-6 was purchased from the American Type Culture Collection (Manassas, VA, USA). The murine pancreatic cancer cell line KPC1 and KPC2 was derived from Kras^LSL−G12D/+^, Trp53^LSL − R172H/+^, and Pdx1-Cre mice. All these cell lines were overexpressed RMRs. Mycoplasma contamination was excluded in these cell lines at the beginning of this study. These cells were cultured in DMEM and RPMI1640 basic medium supplemented with 10% Fetal Bovine Serum (FBS) at 37℃ in a humidified atmosphere of 95% air and 5% CO2.

### Subcutaneous mouse model

The 4-6-week-old BALB/C nude mice were subcutaneously transplanted human cancer cell lines to evaluate chemotherapy and TG-101,209 effects. The murine cancer cells were utilized to establish subcutaneous xenograft in 4-6-week-old C57/B6 mice for immunotherapy assessments. Mice were randomly assigned to each treatment group of six. For the subcutaneous tumor model, the indicated cancer cells of high expression RMRs at a dilution range of 1 × 10^6^ were suspended in a 40 µl PBS and then subcutaneously transplanted into each mouse’s flank. Related drugs were intraperitoneally injected one week later. The observers and recorders in the study were blinded to the grouping. Tumor growth was monitored every three days using a caliper and tumor volumes were calculated by the following formula: Volume = 1/2 L1 × (L2)^2^, where L1 is the length of the long axis and L2 is the length of the short axis. The survival of mice was also recorded at the same time.

### Multiplex fluorescent IHC

The subcutaneous tumors in mice performed into paraffin sectioning were used for immunological assessment of Ki67. Ki67 was labeled by Opal 690(676–694 nm). Isotype controls were used for all assays. Stained slides were scanned over the whole slide using the Vectra Polaris system (PerkinElmer). Phenochart slide reviewer (PerkinElmer) was used to systematically capture tissue heterogeneity in an unbiased manner. The selected images were then captured with a 20× lens using the Vectra Polaris system. Form cell Analysis software 2.4 (PerkinElmer) was used to evaluate the counts of Ki67 positive points per high power field (HPF; 200x). Tumor areas were manually outlined to exclude stromal nuclei. DAPI was used to identify nuclei. Ki67 was then measured in a cell-nucleus-based mode.

### Flow cytometry

The indicated murine cancer cells and T lymphocytes were co-cultured at a ratio of 1:1. The harvested cells were divided into separate tubes for each antibody staining. Add appropriate concentrations of fluorochrome-conjugated antibodies CD8, IFNγ, TNFα, GzmB, perforin, and PD-1 (Table S13), and incubate for 30 min, protected from light. The above samples were detected using a Beckman flow cytometer. The data were analyzed using the software Flow Jo 10.0.

### CD8 + T cells killing assay

The CTLs were used as effector cells and 4T1, LLC1, MC38, Hepa1-6, KPC1, and KPC2 cell lines were used as tumor target cells. The Cell Counting Kit-8 (CCK-8) assay kit was used to conduct a killing assay of CTLs and tumor target cells in vitro. Resuspend tumor target cells at a concentration of 4 × 10^4^/ml and place them on a 96-well cell culture plate, 100µL/well, which means 4,000 cancer cells per well. Resuspend CTLs at a concentration of 4 × 10^4^/ml and place them on a 96-well cell culture plate, 100µL/well, with an effector-target cells ratio of 1:1. Set target cells control group and effector cells control group. After 48 hours of co-cultivation, add 20 µl CCK-8 solution to each well and incubate for 4 hours to detect the absorbance A value of 450. Killing rate calculation = [1- (experimental well absorbance A value - effect control group absorbance A value)/target cells control group absorbance A value] × 100%.

### CCK8 cell viability assay

Cancer cells were seeded in clear, flat-bottom 96-well plates (Corning) at a density of 1000 cells per well. The following day, cancer cells were treated with dilution range of Cyclophosphamide (1.6µM), Adriamycin (1µM), Gemcitabine (5µM), Cisplatin (1.6µM), Oxaliplatin (1µM), Capecitabine (1.6µM), 5-Fluorouracil (0.8µM), TG-101,209 (5µM) or their corresponding combination (5 duplications for groups) for 5 days. The culture media were replaced with fresh RPMI-1640 containing 10% CCK8 and plates were incubated for 3 hours in an incubator. The absorbance was read at 595 nm once a day.

### Drug synergy analysis

We analyzed the synergistic effects of the combination therapy using cell apoptosis assay. Synergy scores were calculated by SynergyFinder ver2 (https://synergyfinder.fimm.fi/). The final synergy scores were interpreted as follows: less than − 10, the interaction between two drugs was likely to be antagonistic; between − 10 and 10, the interaction between two drugs was likely to be additive; and greater than 10, the interaction between the two drugs was likely to be synergistic.

### Statistical analysis

Statistical analysis was generated by R software (v3.6.3) and GraphPad Prism Statistics software version 6.0. For nonnormally distributed variables, we performed the Wilcoxon rank-sum test. For comparisons of more than two groups, Kruskal‒Wallis tests were used as nonparametric methods. The comparison of percentages was by the chi-square test. Student’s t-test was used to compare the mean values. One-way ANOVA was carried out for mouse tumor growth. Each experiment was conducted in triplicate. The data values were presented as the means ± SDs unless otherwise stated. The median survival time was analyzed using Kaplan–Meier curves and the log-rank test was used to analyze differences in the survival time among the different groups. In vivo experimental survival curve, the survival record time of the pancreatic cancer orthotopic transplantation mouse model was 50 days, and the survival record time of other liver transplantation models was 35 days. The end point of the survival record was the death of the animal, and the time of sacrifice of the mouse was the time when the survival curve is finally recorded. The level of statistical significance was defined as * *P* < 0.05, **P < 0.01, *** *P* < 0.001, ****P < 0.0001 and ns, non-significant.

## Results

### Exploring the relationship between RMRs and treatment response in Pan-cancer: insights from single-cell RNA datasets

**Fig. 1:**
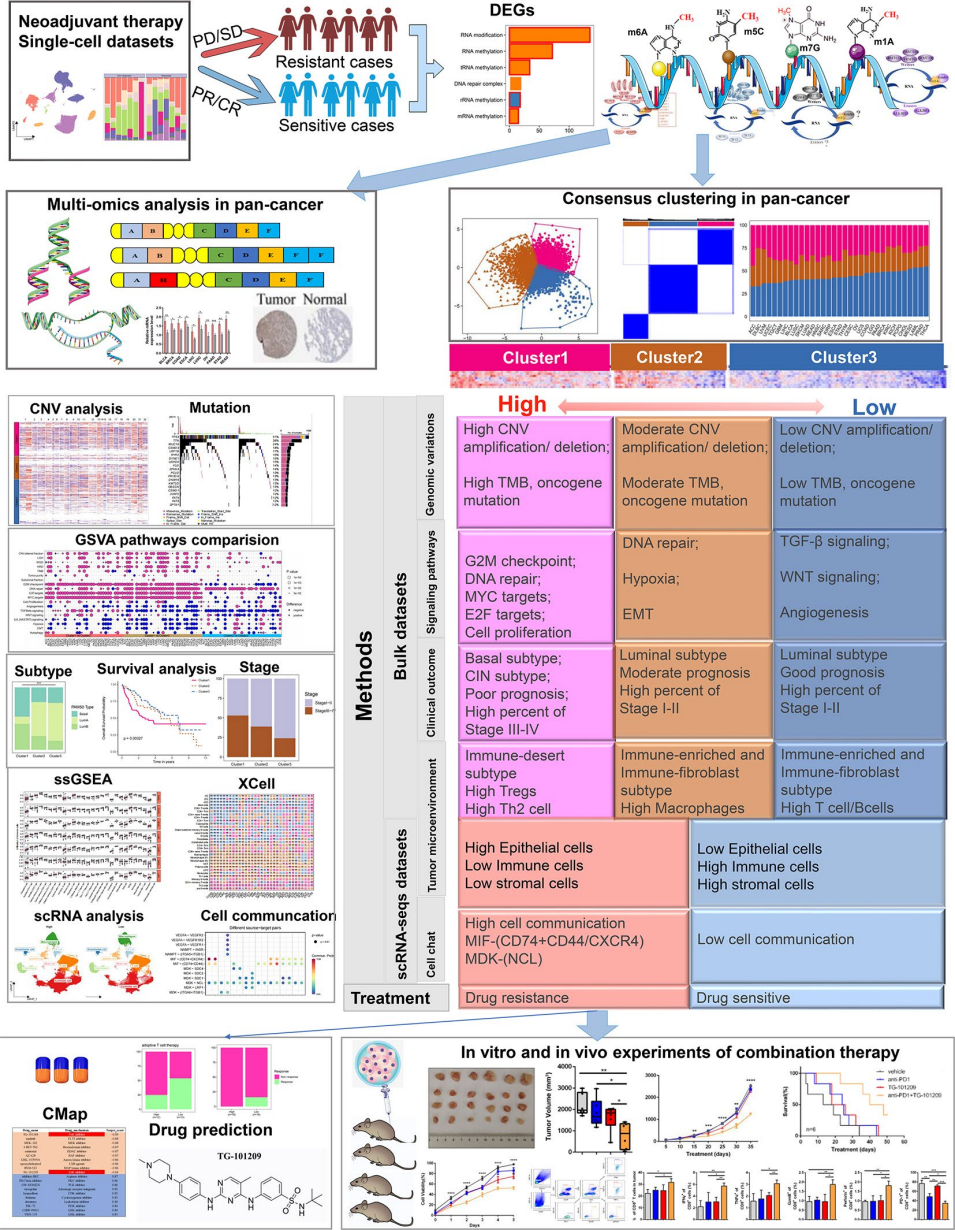
The whole flow chart and the analysis content schematic

The present study outlines our investigation into the relationship between RMRs and cancer treatment response. We conducted an extensive analysis, as illustrated in Fig. 1, which delves into the main findings of our research. To gather data, we curated seven single-cell RNA datasets that encompassed neoadjuvant therapy information. Details of these datasets can be found in Table S1.

**Fig. 2:**
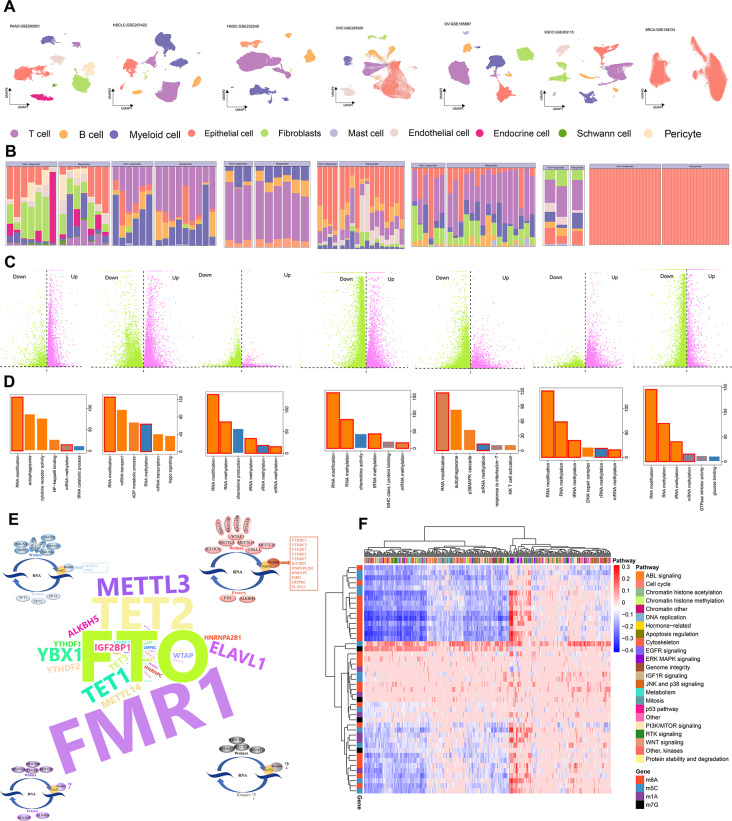
Differences between treatment responders and non-responders are associated with RMRs. (**A**) UMAP plot displaying diverse cell types that have been identified. (**B**) Bar plot showing the percentage of different cell subpopulations in treatment responders and non-responders. (**C**) Volcano illustrating different expression genes between responders and non-responders. (**D**) Demonstrating pathways for differential gene enrichment with *P* < 0.05. (**E**) 46 RNA methylation regulators classified as writers, readers, and erasers from m6A, m5C, m1A, and m7G. (**F**) Heatmap displaying the correlation between RMRs expression and drug IC50 values of the GDSC database

Utilizing established markers (Fig. 2A and Fig. S1), we classified tumor parenchymal and microenvironmental cells into distinct categories, including epithelial cells, T cells, B cells, myeloid cells, mast cells, fibroblasts, endothelial cells, pericytes, a few endocrine cells, and Schwann cells. Notably, except for breast cancer, wherein the analysis focused solely on tumor cells, we observed varying proportions of these cell types between responders and non-responders (Fig. 2B). To further investigate the molecular mechanisms underlying treatment response, we conducted differential expression analysis between the two groups and performed pathway enrichment analysis on the differentially expressed genes. Interestingly, we discovered that these genes consistently participated in RNA methylation modification (Fig. 2C-D). Through an extensive literature search, we identified a total of 46 RMRs, comprising 23 m6A regulators, 6 m1A regulators, 13 m5C regulators, and 4 m7G regulators. Among these, the m6A regulators, particularly FTO and FMR1, have been extensively studied (Fig. 2E, Table S2).

Moreover, we evaluated the expression patterns of these 46 RMRs across approximately 1000 cell lines and found a significant association between their expression levels and drug sensitivity. This crucial finding further highlights the indispensable role played by RMRs in drug therapy (Fig. 2F).

### Comprehensive analysis reveals landscape of genetic and transcriptional aberrations in RMRs across diverse cancer types

**Fig. 3:**
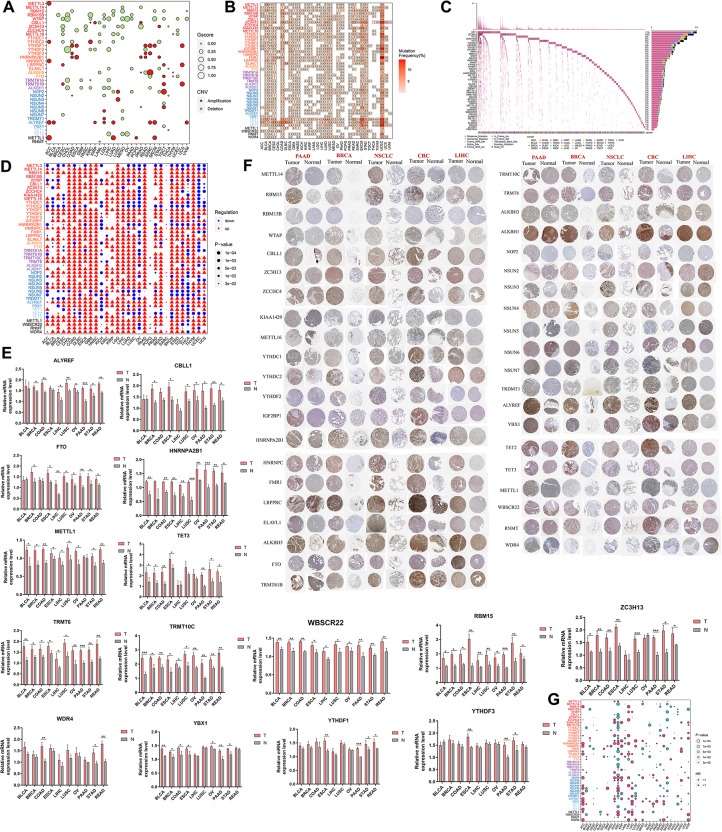
Genomic aberrations, transcriptional alterations, proteomic discrepancy, and survival relevance of RMRs across cancer types. (**A**) Bubble plot showing the Gscore and CNV status of RMRs across cancers. The sizes and colors of the bubbles represent Gscore and CNV status, respectively. (**B**) Heatmap depicting the somatic mutation frequencies of each RMR in each cancer type. (**C**) Waterfall plot showing somatic mutations of RMRS in 2876 tumor samples. The mutation frequencies were shown on the right barplot, and the cancer types of samples were shown at the bottom. (**D**) Dot plot indicating the differentially expressed RMRs between tumor and adjacent normal tissues in each cancer type, with up or down-regulating and the P-value being annotated. (**E**) The bar plots of RT-PCR showing mRNA expression levels of RMRs in tumor and normal tissues (*n* = 6). * *P* < 0.05, **P < 0.01 or *** *P* < 0.001. (**F**) The protein expression of RMRs according to HPA. (**G**) Hazard ratios of overall survival between high and low expression groups regarding each RMRs in each cancer type, with the size and color of the bubble denoting the P-value and Hazard Ratio (HR) of overall survival, respectively

Subsequently, an in-depth analysis of 38 frequently occurring RMRs that showed copy number variations (CNVs) in at least one cancer type was conducted. Notably, we observed that the m6A writer WTAP was located within deletion peaks in 12 cancer types, whereas the m5C reader ALYREF was amplified in 7 cancer types (Fig. 3A). Among a comprehensive dataset of 9991 TCGA samples, we found that m6A writers and readers exhibited the highest frequencies of CNVs (Fig. S2).

Furthermore, the non-silent somatic mutations in RMRs were investigated. These mutations were particularly prevalent in specific cancer types, including skin cutaneous melanoma (SKCM), bladder urothelial carcinoma (BLCA), and uterine corpus endometrial carcinoma (UCEC) (Fig. 3B). Out of the total 9834 pan-cancer samples examined, 2876 (29.24%) samples harbored at least one mutation in an RMR. Interestingly, among these regulators, the m5C erasers displayed the highest mutation frequency across the 2876 samples (Fig. 3C).

To explore the transcriptional levels of RMRs, we compared tumor tissues with adjacent normal tissues obtained from the TCGA and GTEX databases. Despite being frequently affected by CNVs leading to deletions, most RMRs exhibited significant upregulation in tumor tissues. This finding suggests that there is a transcriptional upregulation mechanism governing RMRs in cancers (Fig. 3D). We further validated these findings using RT-PCR, which confirmed the upregulation of most RMRs in tumor tissues across various cancer types (Fig. 3E, Table S3). Additionally, immunohistochemical data from the HPA supported our observations, indicating higher protein levels of RMRs in tumor tissues compared to normal tissues (Fig. 3F).

Moreover, we investigated the prognostic relevance of RMRs by conducting survival analysis across diverse cancer types. Our results revealed that RMRs had a broad impact on cancer survival, with kidney cancers exhibiting a particularly significant association (Fig. 3G). Overall, our comprehensive analysis highlights the crucial role of RMRs in regulating tumor progression.

#### Machine learning constructed distinct regulatory RMR clusters in Pan-cancer and their association with genomic alterations

**Fig. 4:**
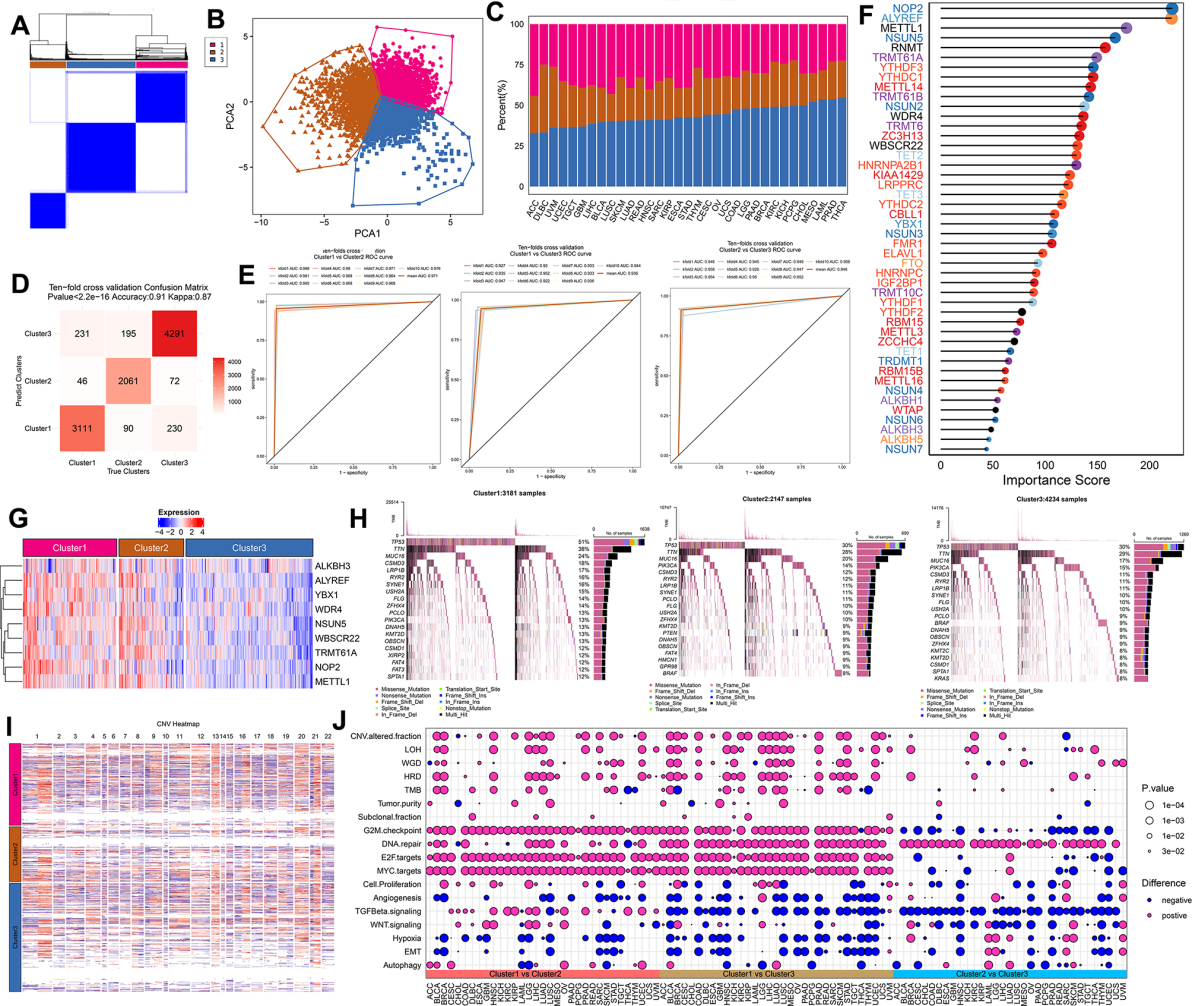
RMRs clusters construction and different genomic characteristics. (**A**) Consensus matrix plot of 46 RNA methylation regulators in pan-cancer. (**B**) Scatter plot of PCA showing distinct three clusters. (**C**) Bar plot showing the percentage of samples of each cluster in each cancer type. (**D**) Confusion matrix of predicted RMRs clusters and true RMRs clusters (**E**) ROC curves of ten-fold cross-validation tests performed on the model built with RMR expression data of the TCGA samples. (**F**) Lollipop plot showing the importance scores of 46 RMRs contributing to the trained random forest model. (**G**) Heatmap of expression levels of 46 RMRs in three clusters. (**H**) Waterfall plot depicting the top 15 genes with the highest mutation rates. (**I**) Heatmap of CNV across all cancer types in three clusters, blue means copy number loss while red means copy number gain. (**J**) Bubble plot visualizing the tumor progression-related features and Hallmark pathways from MSigDB. The size of the bubble denoted the *P*-value. Prune and blue denoted higher and lower pathway enrichment scores in each comparison

Subsequently, a total of over ten thousand samples were classified into three distinct RMR clusters through unsupervised clustering after normalization: Cluster1 (3388 samples), Cluster2 (2346 samples), and Cluster3 (4593 samples) (Fig. 4A, Table S4). The three RMRs clusters were clearly distinguished based on the principal component analysis (PCA) results and the distribution of the clusters varied across specific cancer types, with Cluster3 being the predominant group in most cases (Fig. 4B-C). We utilized the normalized expression matrix of 46 RMRs from TCGA samples to train a random forest model. The predictive accuracy of the model was determined to be 0.91, and the area under the receiver operating characteristic curve in the tenfold cross-validation exceeded 0.9 for the classification of the three pairs of clusters. These results signified that the model demonstrated both accuracy and robustness (Fig. 4D-E). To assess the value of each regulator’s contribution to the model, we calculated its importance score. Notably, the m5C writer NOP2 ranked first among these regulators and was reported to methylate the C(5) position of cytosine 4447 in 28 S rRNA [42] (Fig. 4F). The expression levels of the regulatory RMRs exhibited differential patterns across the different clusters. Specifically, nine regulators displayed a decreasing trend in expression from Cluster1 to Cluster3, indicating a gradient of high to low expression among these clusters (Fig. 4G). Furthermore, their expression profile in the three clusters of individual cancer types resembled the profile of the pan-cancer clusters (Fig. S3). Subsequently, the analysis of three clusters revealed distinct characteristics. Cluster1 displayed a higher frequency of oncogene mutations, particularly TP53, and showed genomic instability with copy number variations. This cluster was also associated with increased genetic alterations and enriched pathways related to cell proliferation and carcinogenic activation, suggesting a more aggressive tumor phenotype (Fig. 4H-J).

### Clinical characteristics and prognosis associated with RMRs clusters across Cancer types

**Fig. 5:**
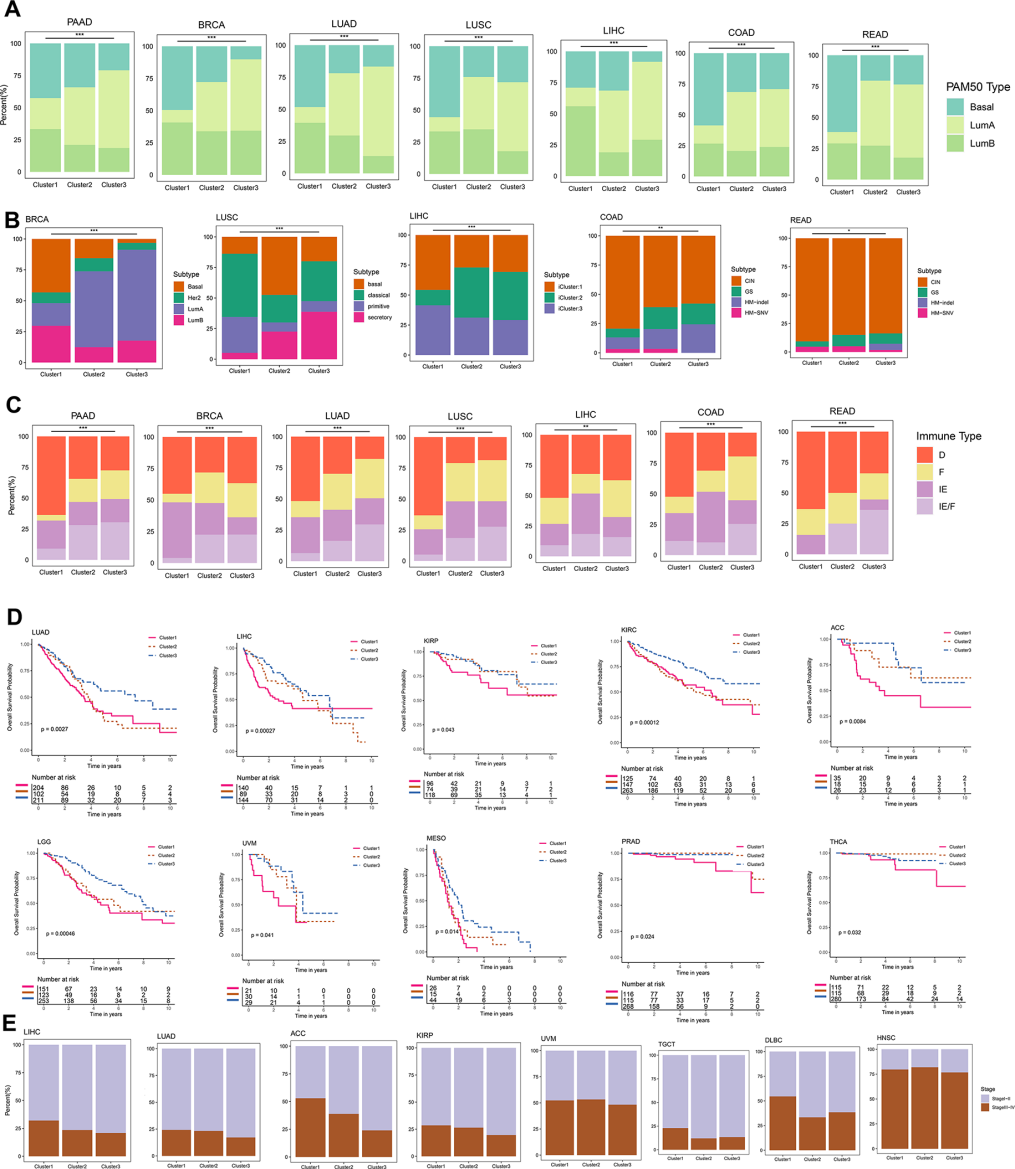
The clinical characteristics of RMRs clusters. (**A**) The proportion of PAM50 subtypes in the RMRs clusters. (**B**) The proportion of typical molecular subtypes in RMR clusters. (**C**) The proportion of immune subtypes in the three RMRs clusters. (**D**) Kaplan-Meier curves showing overall survival in different clusters across 10 cancer types respectively. P-value was calculated by the two-sided log-rank test and *p* < 0.05 was considered statistically significant. (**E**) The fraction of patients with different clinical stages in three RMRs clusters

In addition, the TCGA epithelial tumors were categorized into basal-like, luminal A, and luminal B subtypes using the PAM50 clustering algorithm. In our analysis, we compared the distribution of these subtypes within the three RMR clusters. It was observed that Cluster1 was predominantly composed of the basal-like subtype, while Cluster3 mainly consisted of the luminal A subtype. Furthermore, a comparison of the molecular subtypes revealed that Cluster1 was primarily associated with the basal subtype of breast cancer and the chromosomal instability (CIN) subtype of gastrointestinal tumors (Fig. 5A-B, Table S5-S6).

The TME subtype, including immune-enriched, nonfibrotic (IE), immune-enriched, fibrotic (IE/F), fibrotic (F), and immune-depleted (D), was defined by 29 functional gene expression signatures [43]. Our analysis further demonstrated that Cluster1 showed a strong association with TME subtype D, while Cluster3 was predominantly associated with TME subtypes F and IE/F. This indicates that Cluster1 exhibits an immunosuppressed phenotype resembling the basal-like subtype, which is known to be associated with poor prognosis. Importantly, patients in Cluster1 exhibited significantly worse overall survival in 10 cancer types, poorer disease-specific survival in 12 cancer types, and shorter progression-free survival in 7 cancer types, highlighting the unfavorable prognosis associated with this cluster. Additionally, Cluster1 displayed a higher proportion of stage III-IV cases in 6 cancer types (Fig. 5C-E, Fig. S4A-B, Table S7-8).

#### Link of RMRs clusters and cellular components of the tumor microenvironment in pan-cancer

**Fig. 6:**
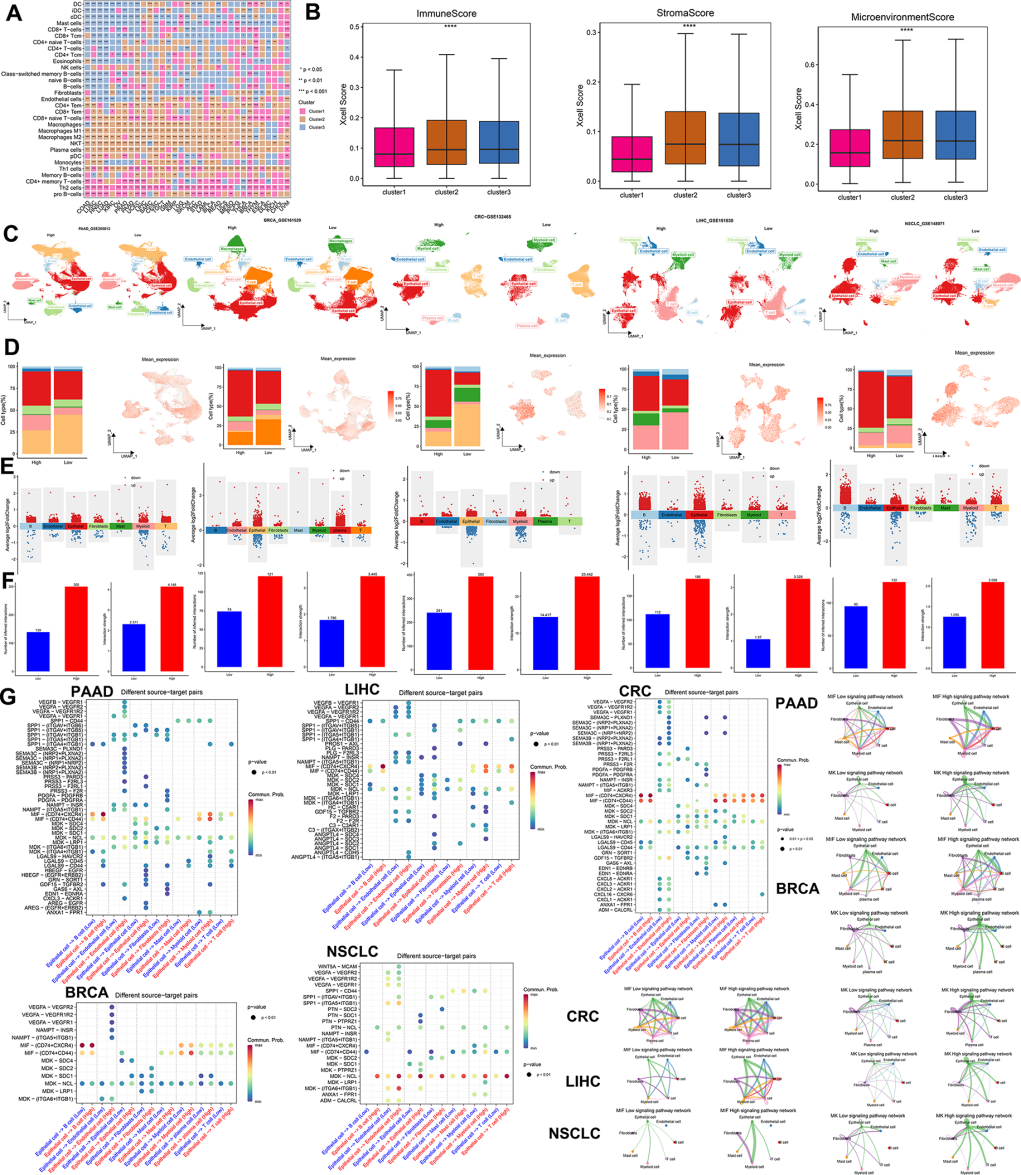
Tumor microenvironment components of RMRs clusters and groups. (**A**) Heatmap showing the abundance scores of immune cell types computed by the xCell algorithm. The color denoted the specific RMR cluster which had a higher abundance score than the other two clusters (**B**) Box plots showing the differences of the immune cell infiltration scores computed by xCell analysis among different clusters. * *P* < 0.05, **P < 0.01, *** *P* < 0.001, ****P < 0.0001 (Kruskal-Wallis test). (**C**) UMAP plot showing annotated cell types that have been defined. (**D**) Bar plot showing the proportion of different cell subpopulations in high-expression and low-expression groups and UMAP plot described the distribution of mean expression values across cell types. (**E**) Characterization of high-expression group and low-expression group differential genes in different cell populations. (**F**) The number of interactions and weight of interaction in high-expression group and low-expression group (**G**) Ligand-receptor interaction pairs genes in epithelial cells and other cell types. MIF and MK signaling pathway networks of high and low groups. The thickness of the interworking line segments represents the number of interworking pairs

On the other hand, the xCell method to analyze TME-infiltrating immune cells in three clusters was utilized. Cluster1 exhibited higher levels of Th2 cells, naive T cells, and pro-B cells, indicating a weaker tumor suppression ability. In contrast, Cluster3 displayed higher levels of CD4 + T cells, CD8 + T cells, NK cells, B cells, cancer-associated fibroblasts, and endothelial cells, suggesting an abundance of tumor-killing effector cells (Fig. 6A, Table S9). ImmuneScore, StromalScore, and MicroenvironmentScore were calculated to assess the immune and stromal components. Cluster3 demonstrated higher immune and stromal cell activity, while Cluster1 had the lowest MicroenvironmentScore (Fig. 6B). Moreover, we used the “ssGSEA” method to evaluate the infiltration of 28 immune cell types in RMRs clusters. Cluster3 showed increased immune cell infiltration, whereas Cluster1 exhibited lower abundance (Fig. S5A, Table S9).

Subsequently, we examined the expression levels of various immune-related genes among the three RMRs clusters. Most genes showed higher expression in Cluster2 and Cluster3 (Fig. S5B-F). By dividing the five single-cell datasets into high and low groups based on the average expression of RMRs, we observed distinct cell annotations through dimensionality reduction clustering, including epithelial cells, immune cells, and stromal cells in five cancer types (Fig. 6C, Fig. S6A). The high-expression group had a higher proportion of malignant tumor cells, while the low-expression group displayed greater T cell infiltration (Fig. 6D). Furthermore, the UMAP plot revealed elevated mean expression in epithelial cells, reinforcing the association between high expression of RMRs and tumor parenchymal cells (Fig. 6D).

Differential gene analysis and pathway enrichment demonstrated that these genes were involved not only in tumor proliferation pathways but also in immune cell differentiation and immune cytokine activity (Fig. 6E, Fig. S6B, Table S10). Cell communication analysis indicated the high-expression group exhibited more frequent and stronger interactions (Fig. 6F). Notably, the reciprocal molecules of macrophage migration inhibitory factor (MIF) and Midkine (MDK) were more pronounced in the high-expression group than in the low-expression group (Fig. 6G).

Based on our analysis, we concluded that high expression of RMRs exhibited characteristics resembling the basal-like subtype, including high proliferation, immune depletion, high malignancy, and poor prognosis. Conversely, low expression of RMRs correlated with increased infiltration of immune cells and large stromal cells, a lower degree of malignancy, and favorable survival outcomes.

#### Decoding the impact of RMR expression on tumor response and unveiling novel therapeutic avenues

**Fig. 7:**
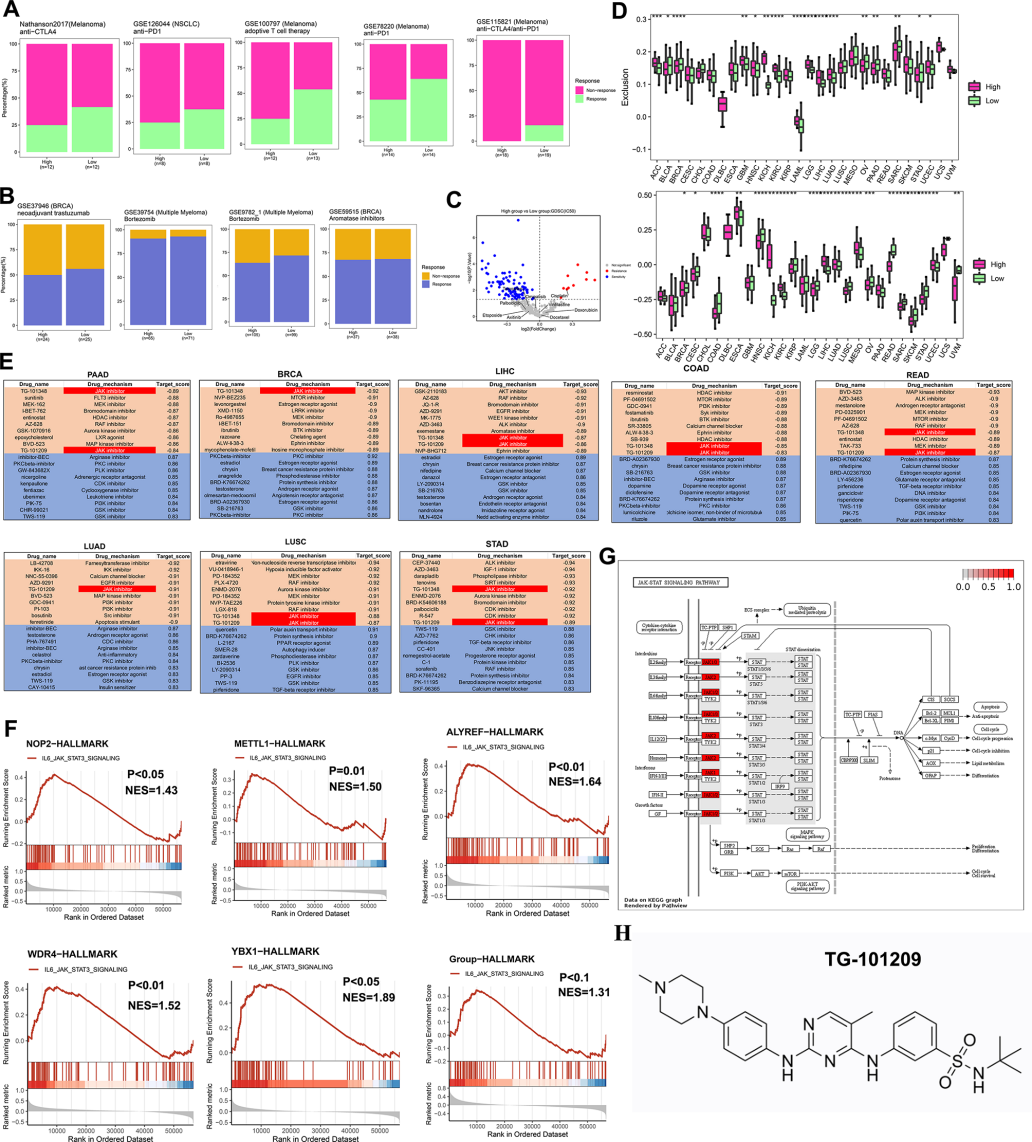
The therapy response of RMRs to drugs. **A.** The fraction of patients with clinical response to immunotherapy. **B.** The fraction of patients with clinical response to chemotherapy. The difference was tested by the chi-square test. **C.** Volcano plot showing drugs that were differentially sensitive in the two groups. **D.** Boxplot revealing dysfunction and exclusion scores in different groups by TIDE methods, in which a higher score means ineffective immunotherapy. **E.** Correlation of RMRs expression with drugs. The table shows the top 10 positively and negatively correlated compounds from the connectivity map. The target score ranged from − 1 (negative connectivity) to + 1 (positive connectivity). **F.** GSEA of RMRs in various cancer types. The enrichment score > 0 demonstrated the positive correlation between RMRs expression and the activity of Hallmark pathways. **G.** Demonstration of JAK-STAT signaling pathways upstream and downstream genes. **H.** Chemical structural formula of JAK inhibitor TG-101,209

Unveiling the impact of RMRs expression on tumor response, we collected multiple datasets of patients who had received immunotherapy or chemotherapy. Upon dividing them into high- and low-expression groups, a significant finding emerged: the high-expression group exhibited a higher proportion of non-responders (Fig. 7A-B). In addition, analysis of IC50 values for various drugs revealed that the high-expression group displayed insensitivity to conventional chemotherapeutic agents (Fig. 7C). We also used the Tumor Immune Dysfunction and Exclusion (TIDE) algorithm [44] to predict immunotherapy response and found the high-expression group showed more immune exclusion scores than the low-expression group (Fig. 7D). Furthermore, in order to evaluate RMRs’ capacity to forecast resistance to chemotherapy and immunotherapy, we compared them with additional characteristics gathered from the published articles. Single-cell dataset of colorectal cancer includes cancer epithelial cells and various microenvironmental cells (Fig. S7A), from which we selected tumor cells to assess the predictive power of RMRs and other genes to predict drug response (Fig. S7B). RMRs were observed to score considerably higher in the non-respond group and to exhibit superior accuracy, AUC, precision, and F1 values when evaluating treatment response. (Fig. S7C-D). Since the scores of RMRs were higher in tumor cell clusters 2 and 4, we designated those clusters as RMRs + epithelial cells and the remaining of epithelial cells as RMRs-epithelial cells, which was in line with the expectation that RMRs + epithelial cells accounted for a higher percentage of non-response patients (Fig. S7E-G). To further explore the relationship between RMRs and immune cells, we did cellular communication between RMRs+/- epithelial cells and other microenvironmental cells. RMRs + epithelial cells were found to have more active MIF, MK, and APP signaling pathways with microenvironment cells than RMRs-epithelial cells. These pathways have been reported to be associated with tumor progression and immunosuppression (Fig. S7H). Similarly, we performed a validation analysis in ovarian cancer and found that RMRs in ovarian tumor epithelial cells were also significantly more potent in predicting treatment response than the genes in other studies (Fig. S7I-K). The RMRs scores of tumor epithelial clusters 1,2,3 were significantly higher than those of cluster 0, so we named clusters 1,2,3 as RMRs + epithelial clusters and the rest as RMRs-epithelial clusters (Fig. S7L-M). Consistent with colorectal cancer, the RMRs + epithelial cluster was concentrated in the non-response patient group (Fig. S7N). Cellular communication of RMRs+/- epithelial cells with other immune or stromal cells was performed, and it was evident that the MIF signaling pathway was more active in RMRs + epithelial cells (Fig. S7O-P). At the same time, we assessed the ability of RMRs and other genes to predict treatment effects at the level of pan-cancer cell lines and bulk samples. Notably, RMRs outperformed other genes in terms of AUC values for predicting the impact of response to immunotherapy and chemotherapy, indicating their superiority in determining therapeutic potential (Fig. S8A-N).

Then, we searched for potential compounds targeting RMRs expression in various cancer types by using connectivity map analysis (CMap) [38], which was employed to reveal functional links between small molecule compounds, genes, and disease states. Notably, the JAK inhibitors, TG-101,209 and TG-101,348 were effective compounds in negatively regulating RMRs expression, while the GSK inhibitor had the opposite effects (Fig. 7E, Table S11). Gene set enrichment analysis (GSEA) of RMRs also revealed positive enrichment in the JAK-STAT pathway (Fig. 7F). Among RMRs, the m5C writer NOP2 contributed the most to distinguishing RMRs clusters and was highly expressed in most cancer types and influenced their survival (Fig. 4F, Fig. S9A-B). We found high expression of NOP2 was related to tumor proliferation and JAK-STAT pathways and also responded to JAK inhibitors in multiple cancer types (Fig. S9C-D). The JAK/STAT pathway is an important cellular cascade that controls a wide range of processes, including cell differentiation, proliferation, and apoptosis [45] (Fig. 7G). The cytokine receptors activate Janus kinases, JAK inhibitors usually target these enzymes to intervene in tumor progression. We therefore hypothesized that TG-101,209, which is a selective JAK2 inhibitor could increase the therapeutic effect in the high expression group (Fig. 7H).

#### Enhancing chemo- and immuno-therapy efficacy in pancreatic cancer with TG-101,209

**Fig. 8:**
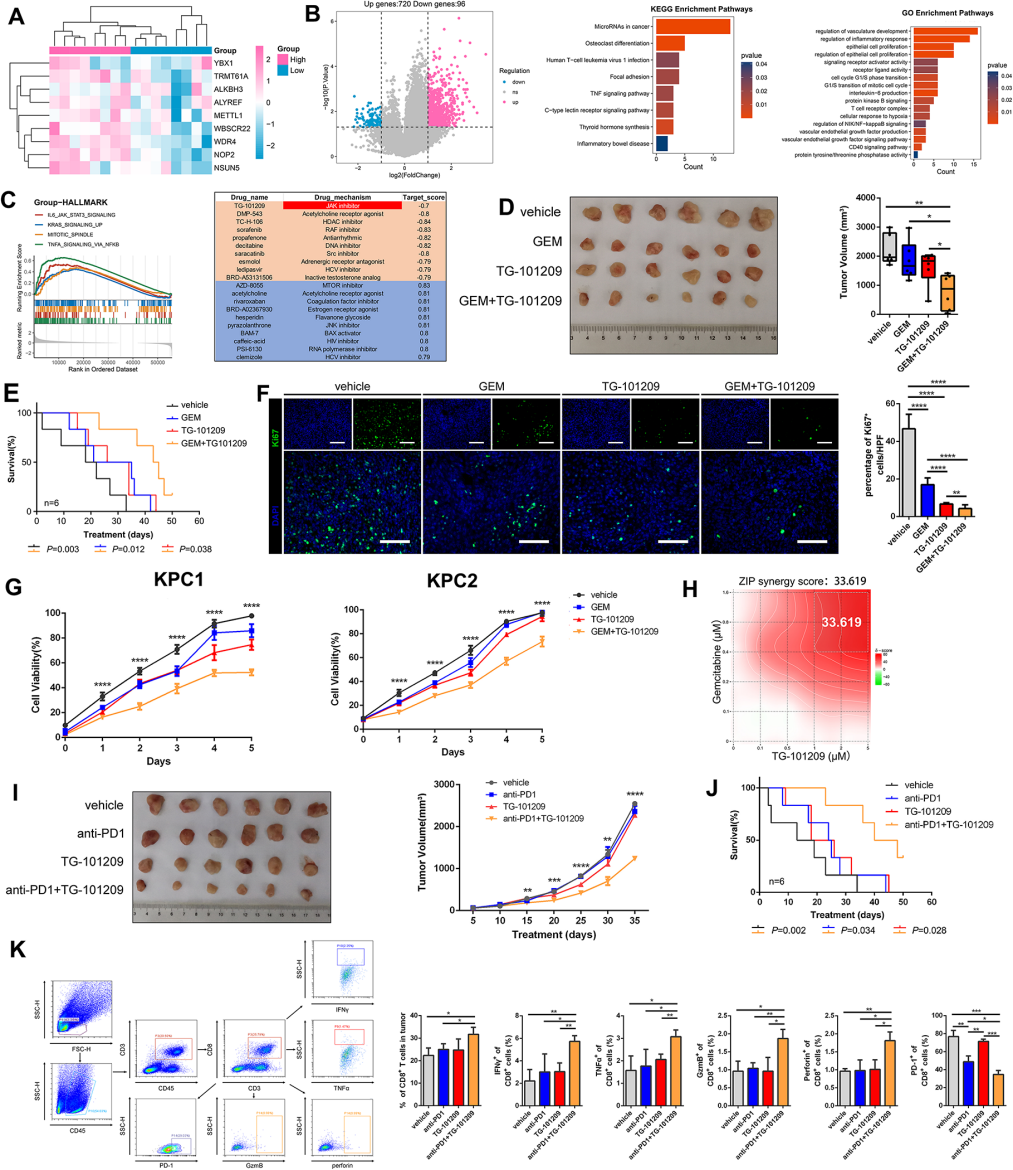
TG-101,209 promoted pancreatic cancer patient response to chemotherapy and immunotherapy. (**A**) The heatmap depicting nine regulators expression of 16 PAAD patients (**B**) Volcano plot describing differential expression genes between high and low groups. Bar plots of KEGG and GO pathways of differential expression genes. (**C**) GSEA analysis of high-expression group and low-expression-group, the pathways were selected under *P* < 0.05. CMap results of predicted potential drugs of high expression of nine regulators. (**D**) Pancreatic tumor cells were subcutaneously transplanted into 4-6-week-old C57/BL mice. The mice were administered GEM, TG-101,209, or GEM combined TG-101,209. Box plot illustrating quantitative comparisons of tumor size across different treatment groups and control groups. (**E**) K-M survival curve of tumor-bearing mice during dosing. Death of mice as the endpoint of the survival record. The transplanted tumor cells were injected into the mouse pancreas in situ. (**F**) The tumors were stripped out from mice, and the frozen sections were stained with anti-Ki67. DAPI staining was included to visualize the nuclei. (**G**) Line graph telling proliferation ability in different treatment groups of KPC1 and KPC2 cell lines. (**H**) Heat map displaying the interaction of GEM and TG-101,209. (**I**) Mice were subcutaneously injected with pancreatic tumor cells. Tumor-bearing mice were injected with anti-PD1, TG-101,209, or a combination of anti-PD1 and TG-101,209. Tumor growth curve showing growth rate and volume size until mice sacrificed. (**J**) Survival curves of orthotopic pancreatic tumor-bearing mice in control, anti-PD1, TG-101,209, and combined therapy groups. The time at which the mice were sacrificed was the time at which the survival curve was finally recorded. **K.** Flow cytometry sorting CD8 + T cell types and the mathematical statistics were made in CD8 + T cells, effector CD8 + T cells, and exhausted CD8 + T cells of different immunotherapy groups

To gain deeper insights into the progression of pancreatic tumors, we conducted a comprehensive study involving 16 pancreatic cancer patients, we examined the impact of RMRs on tumor development (Fig. 8A, Table S12). Patients were divided into high and low RMR groups to investigate the DEG patterns related to cell cycle regulation, proliferation, and immune modulation (Fig. 8B). Our analysis identified 816 DEGs between the two groups, shedding light on the molecular alterations associated with RMR-mediated pancreatic tumor progression. Notably, the high-expression group showed a significant association with the JAK-STAT signaling pathway, suggesting the potential of using the JAK inhibitor TG-101,209 as a targeted therapy for these patients (Fig. 8C).

Subsequently, our in vivo and in vitro experiments demonstrated a significant reduction in tumor volume when TG-101,209 was administered in combination with GEM (Gemcitabine), a conventional chemotherapy drug. Moreover, the combination therapy resulted in improved overall survival compared to the use of either drug alone (Fig. 8D-E). Complementing these findings, mIHC analysis revealed a notable decrease in Ki67 levels, a well-established marker of cellular proliferation, within the combination chemotherapy group (Fig. 8F). Additionally, CCK-8 assays demonstrated that co-medication effectively suppressed tumor cell vitality, particularly in pancreatic cancer cell lines (Fig. 8G). Notably, our analysis revealed a strong synergistic effect with a ZIP Synergy score of 33.619 between GEM and TG-101,209, further supporting the potential clinical utility of this combination therapy (Fig. 8H).

We also investigated the sensitizing effect of TG-101,209 in combination with immunotherapy. Monotherapy using PD1 blockade alone displayed limited efficacy in tumor elimination; however, when combined with TG-101,209, it exhibited enhanced effectiveness (Fig. 8I). Co-administration of TG-101,209 and PD1 blockade significantly extended the survival of mice bearing experimentally induced tumors (Fig. 8J). Furthermore, flow cytometric analysis revealed that combination immunotherapy augmented the number of CD8^**+**^T cells and effector CD8^**+**^T cells secreting cytotoxic proteins while reducing the population of exhausted CD8^**+**^PD1^**+**^T cells (Fig. 8K).

In conclusion, these findings provide valuable insights into the treatment strategies for pancreatic cancer, suggesting the potential utility of targeting RMRs as a therapeutic approach to optimize patient outcomes.

### TG-101,209 sensitized high-RMRs-expressing tumors to chemotherapy across cancer types

**Fig. 9:**
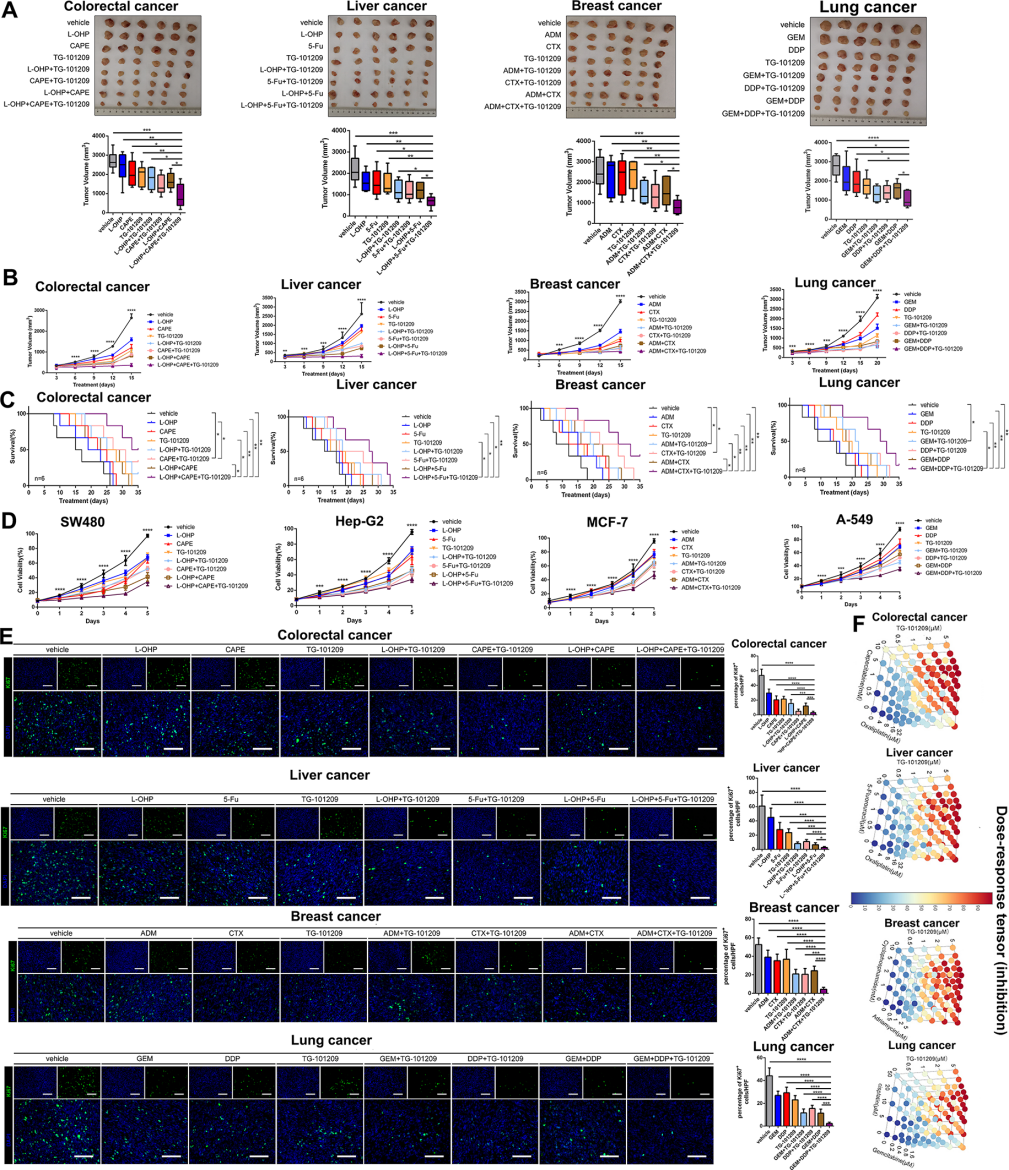
TG-101,209 increased tumor response to chemotherapeutic agents. (**A**) Subcutaneous growth of mice in the control group, single-drug, two-drug combination, and TG-101,209 in combination with two traditional drugs groups (*n* = 6). (**B**) The volume change of nude mice implanted tumors was recorded every 3 days. Each bar represents the mean ± SD for six animal measurements. (**C**) The K-M survival analysis in different chemotherapy drug treatment groups was performed until the sacrifice of mice. Tumor cell lines were transplanted to the liver in situ of mice. (**D**) CCK-8 assay was performed to determine the cell viability after treatment in MCF-7, A-549, SW480, and Hep-G2 cell lines. (**E**) Representative ki67 and 4-6‐Diamidino‐2‐phenylindole (DAPI) immunofluorescence staining of tumor sections from the tumor tissues in different treatment groups. (**F**) Three-dimensional stereo thermograms showing synergistic promotion between two-by-two drugs. * *P* < 0.05, **P < 0.01 or *** *P* < 0.001

We further investigated the antitumor activities of TG-101,209 and other chemotherapeutic drugs in various cancer cell lines with high-expression RMRs, including colorectal, liver, breast, and lung cancers (Fig. 9A). Co-administration of TG-101,209 with L-OHP (Oxaliplatin) or CAPE (Caffeic acid phenethyl ester) showed the most effective tumor regression in colorectal cancer compared to mono- or dual-drug treatments (Fig. 9A). Similarly, in liver, breast, and lung cancers, combinations of TG-101,209 with 5-FU (5-Fluorouracil), L-OHP, ADM (Adriamycin), CTX (Cyclophosphamide), GEM, or DDP (Cisplatin) resulted in smaller tumor sizes compared to mono-chemotherapy groups (Fig. 9A).

Combination therapy significantly improved the survival of tumor-bearing mice and suppressed tumor growth during treatment (Fig. 9B-C). In vitro analysis using CCK-8 assay demonstrated that combined therapy effectively disrupted tumor cell viability (Fig. 9D). Additionally, DAPI and Ki67 immunofluorescence staining of tumors implanted in mice showed a significant reduction in cell proliferative activity with the combination therapy (Fig. 9E). Evaluation of combinatory effects using SynergyFinder revealed strong synergistic effects between traditional chemotherapeutic agents and TG-101,209 (Fig. 9F).

We examined the protein levels of JAK signaling pathway genes in the TG-101,209-treated and control groups and the activated JAK1/JAK2/STAT1/STAT3 proteins levels were lower in the treated cell lines, suggesting that TG-101,209 specifically inhibits the proteins of the JAK-STAT signaling pathway (Fig. S10A). By comparing the expression levels of RMRs genes in the blank control group and the TG-101,209 treatment group, we found that in the absence of other interfering agents, TG-101,209 significantly decreased the mRNA levels of RMRs genes. However, if the drug HJC0146, an inhibitor of the total JAK-STAT pathway was added in advance, the effect of TG-101,209 in decreasing the expression of RMRs was not obvious. Therefore, TG-101,209 functioned as a specific inhibitor of the JAK-STAT signaling pathway thereby reducing the transcriptome level of RMRs (Fig. S10B-J).

### TG-101,209 sensitized high-RMRs-expressing tumors to Immunotherapy across cancer types

**Fig. 10:**
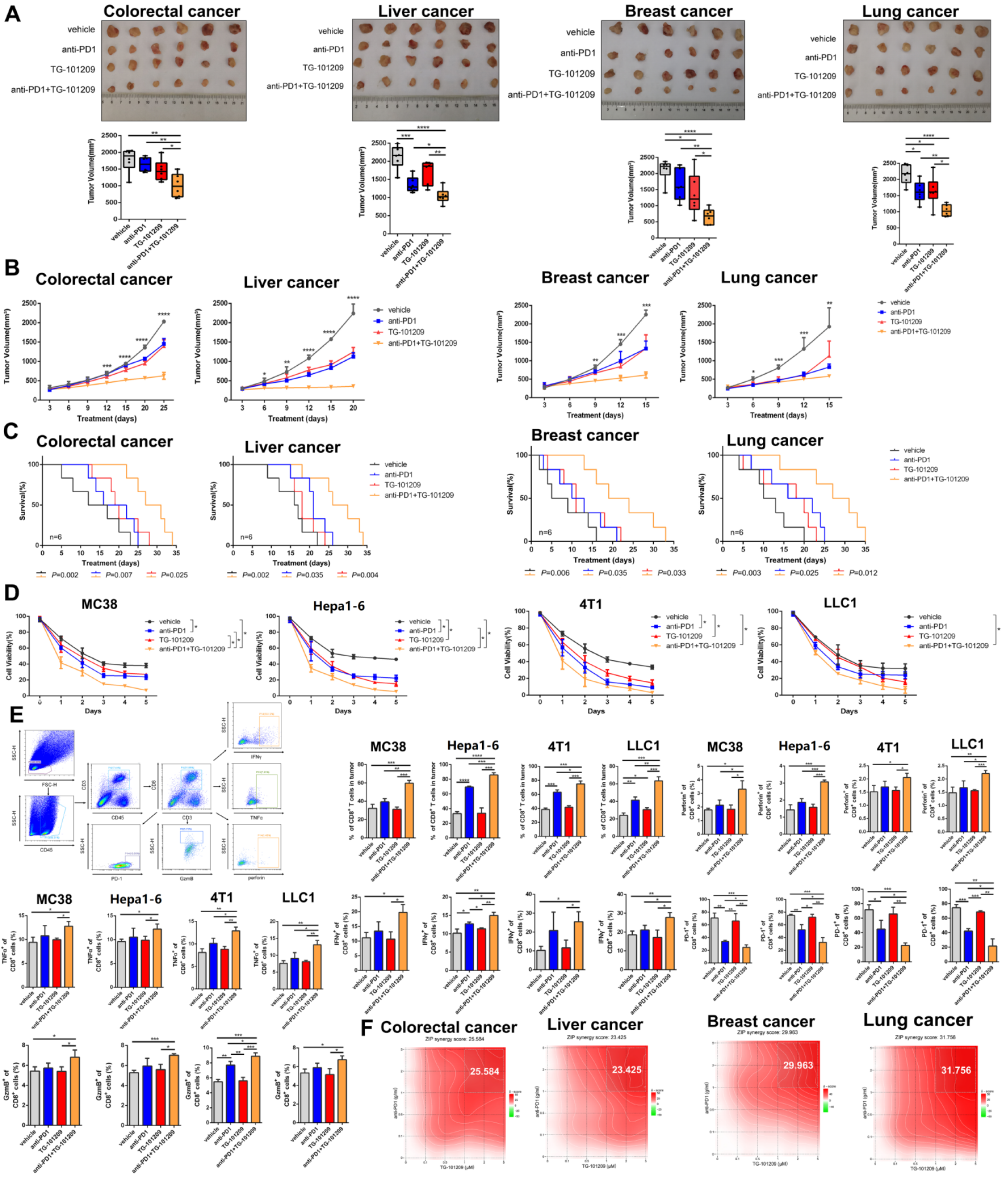
TG-101,209 enhanced tumor response to immunotherapy. (**A**) The tumor size and volume were documented in anti-PD1, TG-101,209, two-drug combination groups, and control groups. (**B**) The tumor growth curve was recorded every three days during the period of treatment. Each bar represents the mean ± SD for six animal measurements. (**C**) The survival time curve of tumor-bearing mice in different treatment groups. Death of mice as the endpoint of the survival record. Tumor cell lines were injected into the orthotopic liver of mice. (**D**) Cell viability curve of different immunotherapy groups. (**E**) Flow cytometry analysis was performed in the harvested tumors; Representative dot plots and bar plots of the percentage of CD8 + T cells, CD8 + TNFα + T cells, CD8 + IFNγ + T cells, CD8 + PD1 + T cells, CD8 + perforin + T cells and CD8 + Grzmb + T cells. (**F**) The heat map shows the synergistic effect of the anti-PD1 and TG-101,209. ZIP Synergy scores > 10 indicate synergism (red regions), scores < -10 indicate antagonism (green regions), and scores between − 10 and 10 mean the interaction between two drugs was likely to be additive. * *P* < 0.05, **P < 0.01 or *** *P* < 0.001

Furthermore, we observed that the combination of anti-PD1 and TG-101,209 had a greater impact on reducing tumor volume and prolonging the survival of mice with tumors compared to anti-PD1 or TG-101,209 alone (Fig. 10A-C). The combination therapy also resulted in greater inhibition of tumor cell viability (Fig. 10D). Flow cytometry analysis of harvested tumors indicated reduced infiltration of CD8^**+**^PD1^**+**^T cells but improved ratios of CD8^**+**^T cells and increased functional factors (IFNγ, TNFα, GZMB, and perforin) within CD8^**+**^T cells (Fig. 10E). We evaluated the magnitude of mutual reinforcement between anti-PD1 and TG-101,209, showing strong ZIP synergy scores (Fig. 10F).

By evaluating the contribution of immune cells, such as effector CD8 + T cells, B cells, NK cells, regulatory T cells (Tregs), and myelosuppressive cells, to immune activation and clinical prognosis in tumor patients in the TCGA pan-cancer dataset, we were able to derive coefficients for each immune cell. This allowed us to further evaluate the relationship between TG-101,209 and immune microenvironment activation. (Fig. S11A). We further combined the weighted model coefficients of the number of infiltrations of each immune cell in the experimental mouse model to evaluate the TME-activated scores in the TG-101,209 group and the control group. In multiple cancers, the high immune score group had a higher proportion of the group treated with TG-101,209, and the immune activation function of the treatment group was also higher than the control group. The TME-activated score can well predict the TG-101,209 treatment group and control group with high AUC of the area under the ROC curve (Fig. S11B-F).

Overall, the combination therapy with TG-101,209 enhanced the killing function of CD8^**+**^T cells, reduced exhausted CD8^**+**^T cells, and decreased tumor burden in high-RMRs-expressing tumors. These findings highlight the potential of combined TG-101,209 therapy as an effective approach for improving treatment outcomes in various cancer types.

## Discussion

Cancer has a high rate of lethality, recurrence, and metastasis, becoming the greatest threat to human health. Surgery, chemotherapy, radiotherapy, and immunotherapy have stepped up as the mainstay of treatment modalities. But the progression, recurrence, and metastasis of cancer still haven’t been curbed, the most important reason is tumor drug resistance. The single-cell datasets of chemotherapy and immunotherapy suggested that RNA methylation was associated with neoadjuvant therapy resistance. We reviewed the publicly available literature and found 46 recognized regulators of RNA methylation, involving m6A, m5C, m1A, and m7G. Multi-omics and RT-PCR experiments demonstrated that RMRs were genomically engineered in tumors with increased transcript levels and protein levels and were associated with poor prognosis in pan-cancer. RNA methylation is one of the epigenetic modifications and is widely observed in prokaryotes and eukaryotes. It has been shown to take an important role in tumor progression, tumor angiogenesis, and tumor drug resistance [46]. Increasing evidence has suggested that RNA methylation pathways were misregulated in human cancers and would be ideal targets for cancer therapy [47].

Through random forest machine learning, we identified three distinct RMRs clusters in pan-cancer and explored their difference in genomic variation, molecular mechanism, TME characteristics, clinical subtypes, and survival performance. Cluster1 exhibited characteristics of more genomic variations, high proliferation, immune depletion, and poor prognosis. Cluster3 was associated with a high degree of immune cell enrichment, a lower degree of malignancy, and a good prognosis. As an intermediary, Cluster2 had moderate immune cell infiltration, an intermediate degree of malignancy, and a relatively good survival performance. The Cluster1 with high-expression RMRs showed striking features of T cell absence or exclusion, which are called “cold” tumors, while hot tumors are infiltrated by T cells and represented by molecular signatures of immune activation [48]. Nine regulators of 46 RMRs in Cluster1, Cluster2, and Cluster3 showed a trend of high medium, and low expression and they were more important in the random forest model according to contribution score. Therefore, the expression of nine RMRs was more representative of overall RMRs performance in tumors.

We grouped single cells high and low based on nine RMRs expression to discover the impact of RMRs on the tumor microenvironment in various cancer types. Consistent with the results of bulk data analysis, the high-expression group had more tumor parenchymal cells while the low-expression group had more immune cells and stromal cells. Several articles more or less described RNA methylation factors remodeling the tumor microenvironment in single-cell analysis. scRNA-seq identified decreased myeloid-derived suppressor cells, coexisting with increased cytotoxic T cells in YTHDF1 knockout tumors [49]. The m6A methylation reader IGF2BP2 activated endothelial cells to promote lung adenocarcinoma angiogenesis and metastasis [50]. Cellular communication indicated the high-RMRs- expressing tumor epithelial cells had more interactions with microenvironment cells and the receptor-ligand pairs were more in the MDK and MIF signaling pathways. It has been verified that MDK reconstructed the immunosuppressive environment in melanoma and gallbladder cancer [51, 52]. Inhibition of MIF-CD74 signaling promoted CD8 + T cell infiltration and drove macrophage conversion to pro-inflammatory M1 macrophages in the TME [53].

**Fig. 11:**
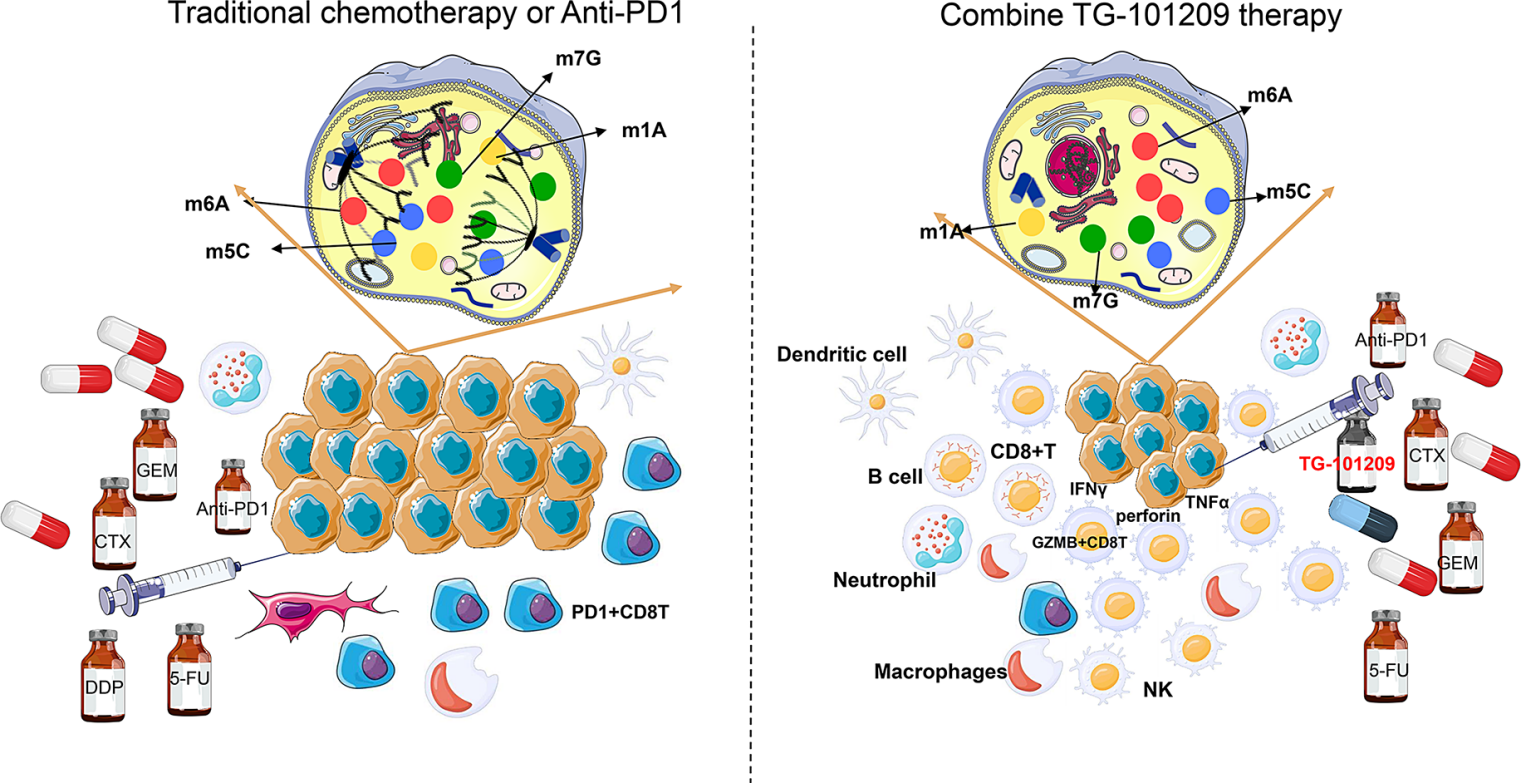
Proposed model for the role of TG-101,209 in increasing the sensitivity to chemotherapy and immunotherapy. (Left) Overexpressed RMRs induced tumor proliferation, immune suppression, and treatment resistance. (Right) The addition of TG-10,129 decreased tumor growth, increased effector immune cells, and promoted therapy responsiveness

The malignant behavior of high-RMRs-expressing tumors compels us to find effective drugs for their treatment. We collected representative datasets with information on drug treatment effects and evaluated the sensitivity of RMRs subtypes to chemotherapy and immunotherapy. The high-RMRs-expressing tumors were insensitive to conventional chemotherapeutic agents and immunotherapy inhibitors. Based on RMRs’ molecular characterization and *in vitro/in vivo* pan-cancer experiments, we predicted potential therapeutic target drug JAK inhibitor TG-101,209. TG-101,209 was a small molecule JAK2-selective kinase inhibitor, which had been proven to induce cell cycle arrest and apoptosis in the human JAK2V617F-expressing acute myeloid leukemia cell line [54]. It had been reported that both RMRs and JAK inhibitors were strongly associated with programmed tumor cell death. Methylation modification of m6A in hepatocellular carcinoma can regulate the iron death program to modulate tumor progression [55]. RNA modification of METTL17 in mitochondria of colorectal carcinoma leads to decreased iron death activity in tumor cells and promotes tumor proliferation [56]. JAK inhibitor promotes the death of myeloma cell lines and inhibits the growth of myeloma cells [57]. JAK inhibitors combined with DNMT1 inhibitors can promote cervical cancer tumor cells to undergo apoptotic cell death [58]. Compared with the control group and the monotherapy group, the tumors in the combined TG-101,209 treatment group were significantly smaller, the proliferation rate and activity of tumor cells were reduced, and there was a significant increase in functional CD8 + T cells and a substantial reduction in CD8 + PD1 + T cells (Fig. 11). A previously reported study also described JAK inhibitors combined with other anti-inflammatory or immunomodulatory agents achieved maximum treatment effect with fewer adverse events [59]. Pancreatic cancer specimens from our cohort, which is called the king of cancers with poorly treated, were collected to demonstrate that high-RMRs-expressing tumors were associated with JAK-STAT pathway activity and can be TG-10,129 targeted. In vitro/in vivo experiments validated that pancreatic cancer was better treated with GEM or anti-PD1 in combination with TG-101,209. Now cytotoxic chemotherapy is still the mainstay of treatment for most pancreatic cancer patients, while immune checkpoint inhibitors and other therapies have no utility for most patients [60].

In summary, our study highlighted that RMRs were widely associated with chemoimmunotherapy resistance in pan-cancer single-cell data. For the first time, we integrated all RNA methylation regulators and performed multi-omics analysis and machine learning to construct precise stratified subtyping of RMRs for cancer therapeutic guidance. RMRs subtypes were related to tumor progression, immune cell infiltration, patients’ prognosis, and treatment responsiveness in pan-cancer. High-RMRs-expressing tumors were insensitive to tumor chemotherapy/immunotherapy. We predicted and experimentally demonstrated the co-administration of JAK inhibitor TG-101,209 could enhance high-RMRs-expressing tumors’ sensitivity to conventional chemotherapy and immunosuppressants in multiple cancer types. However, our study has some defects, we don’t have enough research on drug mechanisms and we need more multi-center clinical drug trials for compensating the limitations of animal models. The connection between RMRs and the JAK-STAT signaling pathway was explored and verified, but the association of other signaling pathways with RMRs was not investigated in depth. In the immunotherapy combined with the JAK inhibitor regimen, we only chose anti-PD1 and did not incorporate other immunotherapeutic agents for in vivo experimental validation.

### Electronic supplementary material

Below is the link to the electronic supplementary material.

Supplementary Material 1

Supplementary Material 2

Supplementary Material 3

Supplementary Material 4

Supplementary Material 5

Supplementary Material 6

Supplementary Material 7

Supplementary Material 8

Supplementary Material 9

Supplementary Material 10

Supplementary Material 11

Supplementary Material 12

Supplementary Material 13

Supplementary Material 14

Supplementary Material 15

Supplementary Material 16

Supplementary Material 17

Supplementary Material 18

Supplementary Material 19

Supplementary Material 20

Supplementary Material 21

Supplementary Material 22

Supplementary Material 23

Supplementary Material 24

Supplementary Material 25

## Data Availability

The datasets used during the current study are available from the corresponding author upon reasonable request.
