# Supplementary figures and images for: Targeting treatment resistance: unveiling the potential of RNA methylation regulators and TG-101,209 in pan-cancer neoadjuvant therapy
Source: J Exp Clin Cancer Res. 2024 Aug 19;43:232. doi: 10.1186/s13046-024-03111-x (PMC11331809; doi:10.1186/s13046-024-03111-x)

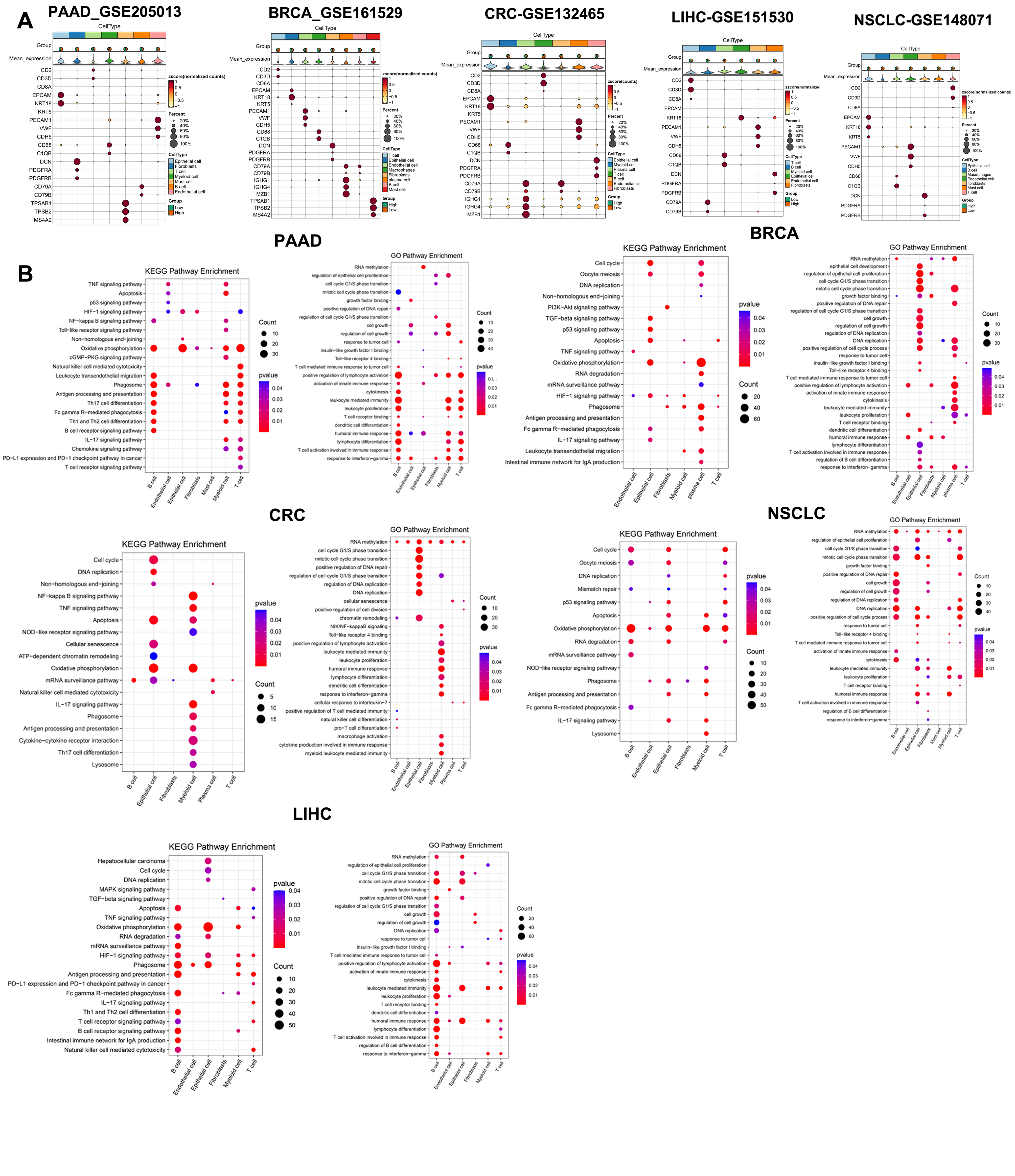

Supplement: Supplementary file 1 — Supplementary Material 1 [file 13046_2024_3111_MOESM1_ESM.tif]

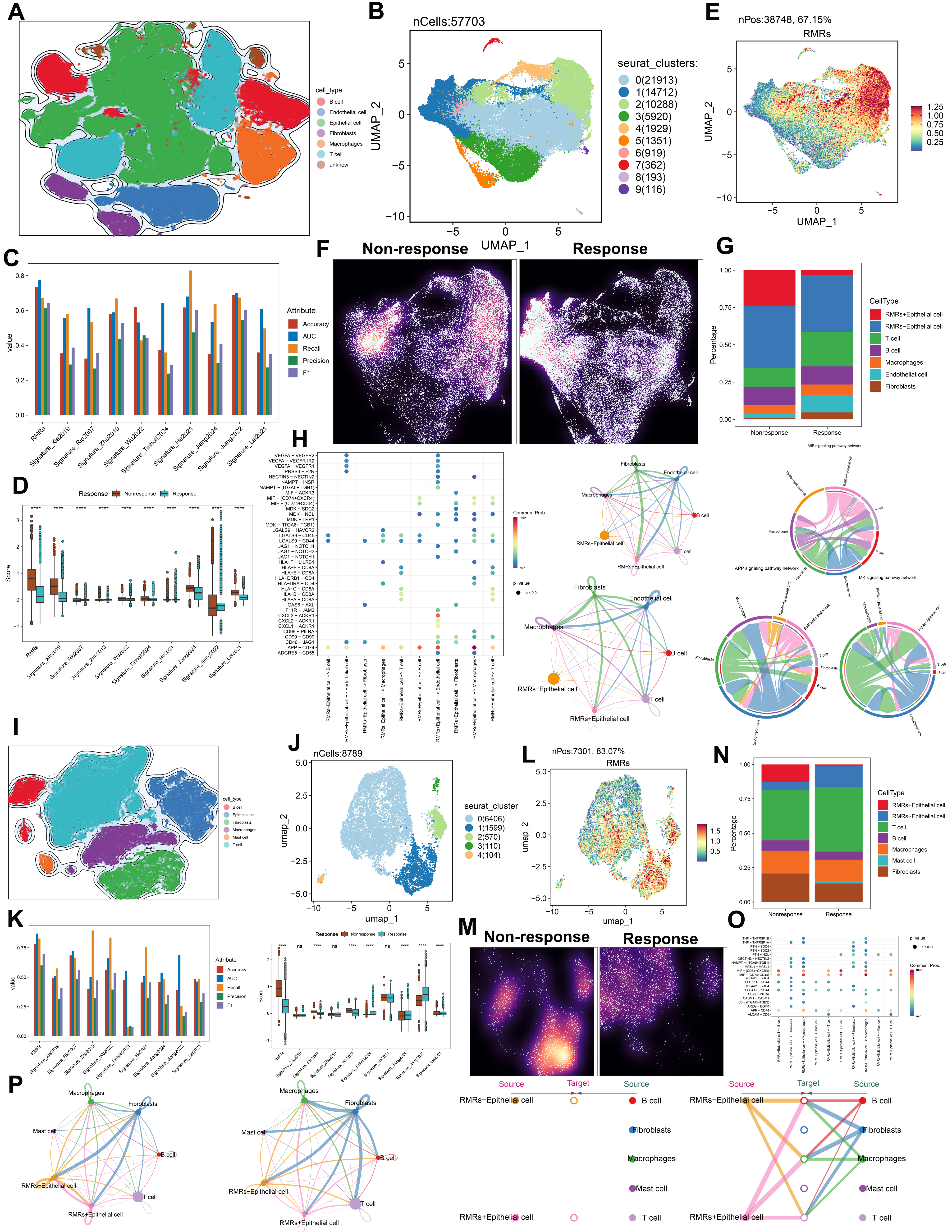

Supplement: Supplementary file 2 — Supplementary Material 2 [file 13046_2024_3111_MOESM2_ESM.tif]

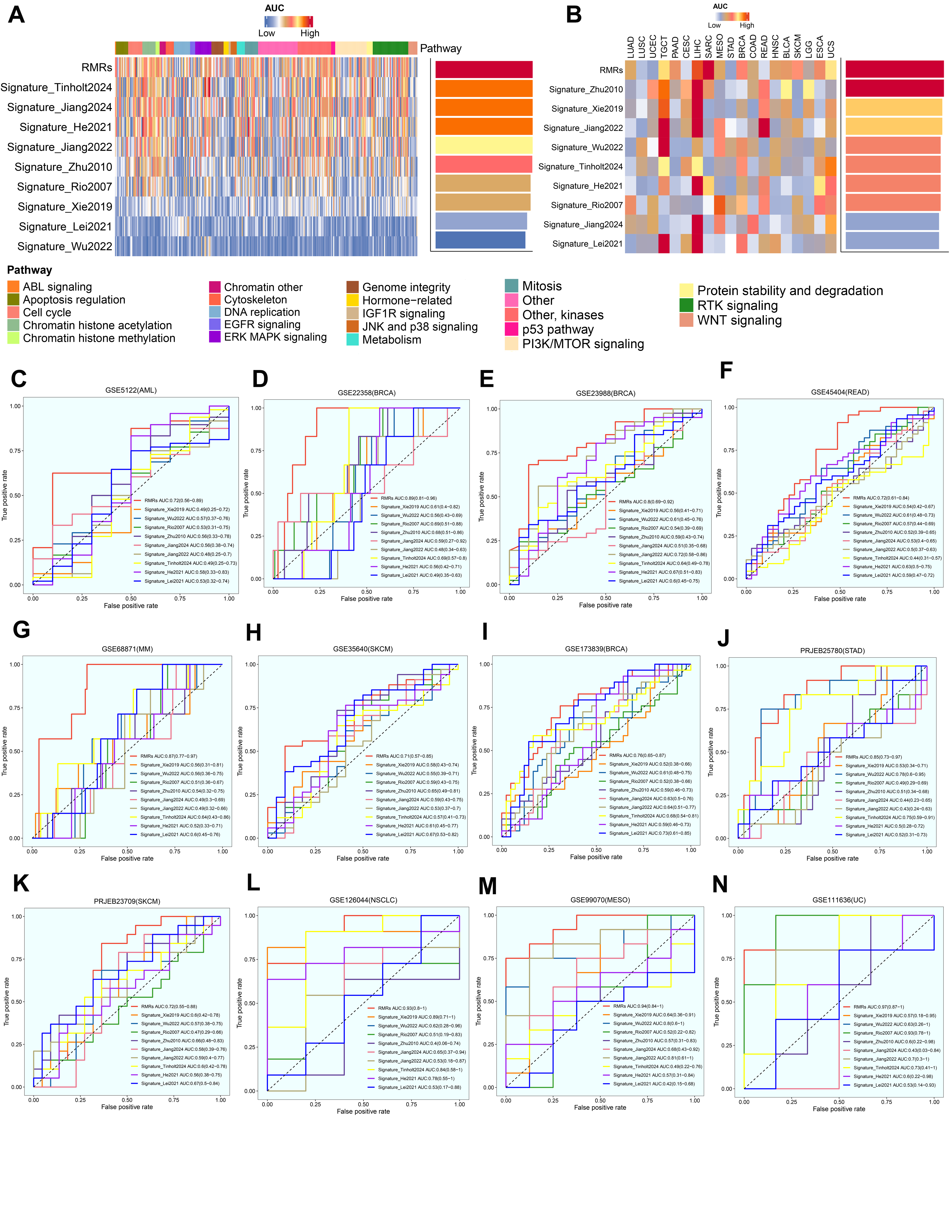

Supplement: Supplementary file 3 — Supplementary Material 3 [file 13046_2024_3111_MOESM3_ESM.tif]

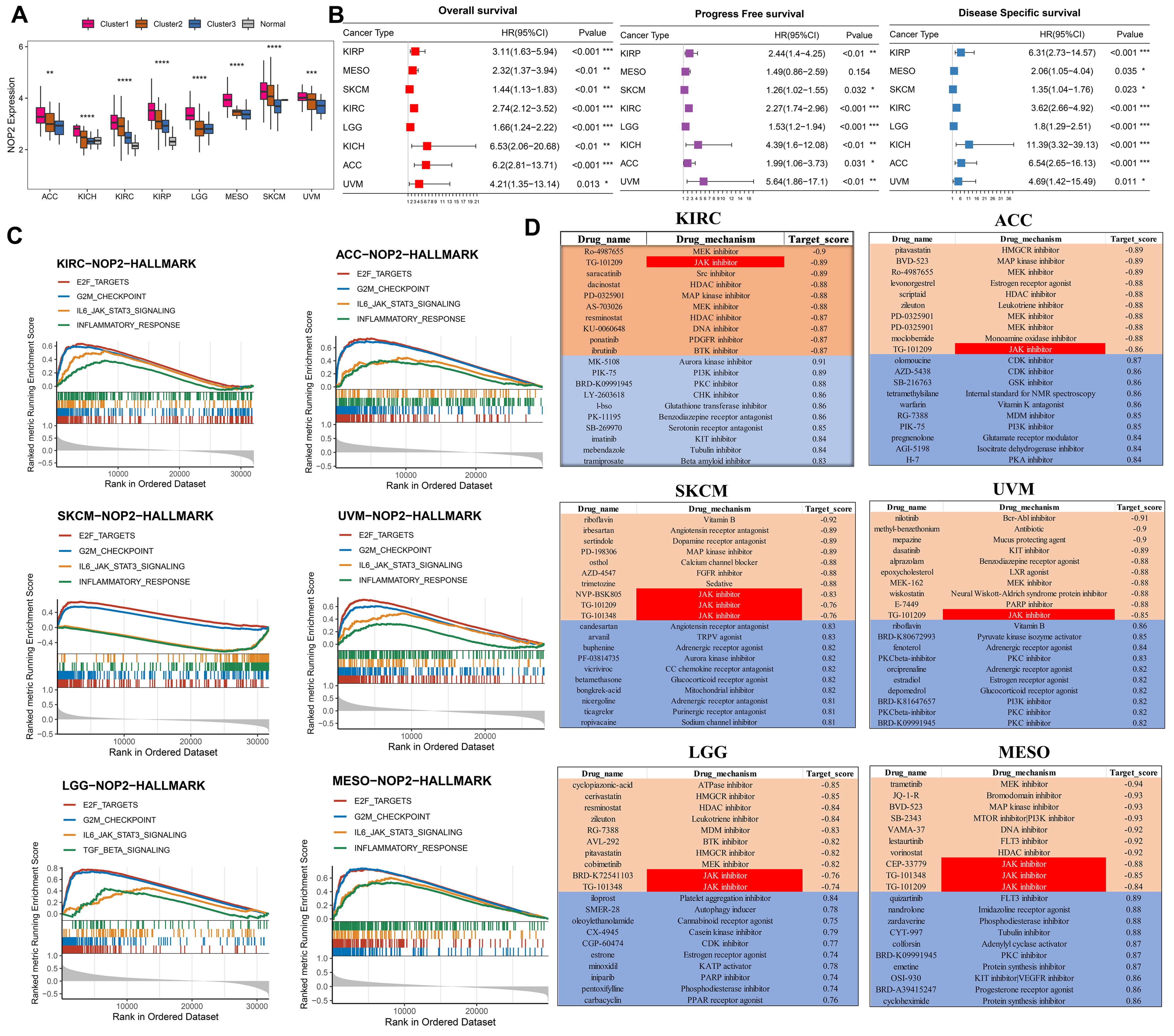

Supplement: Supplementary file 4 — Supplementary Material 4 [file 13046_2024_3111_MOESM4_ESM.tif]

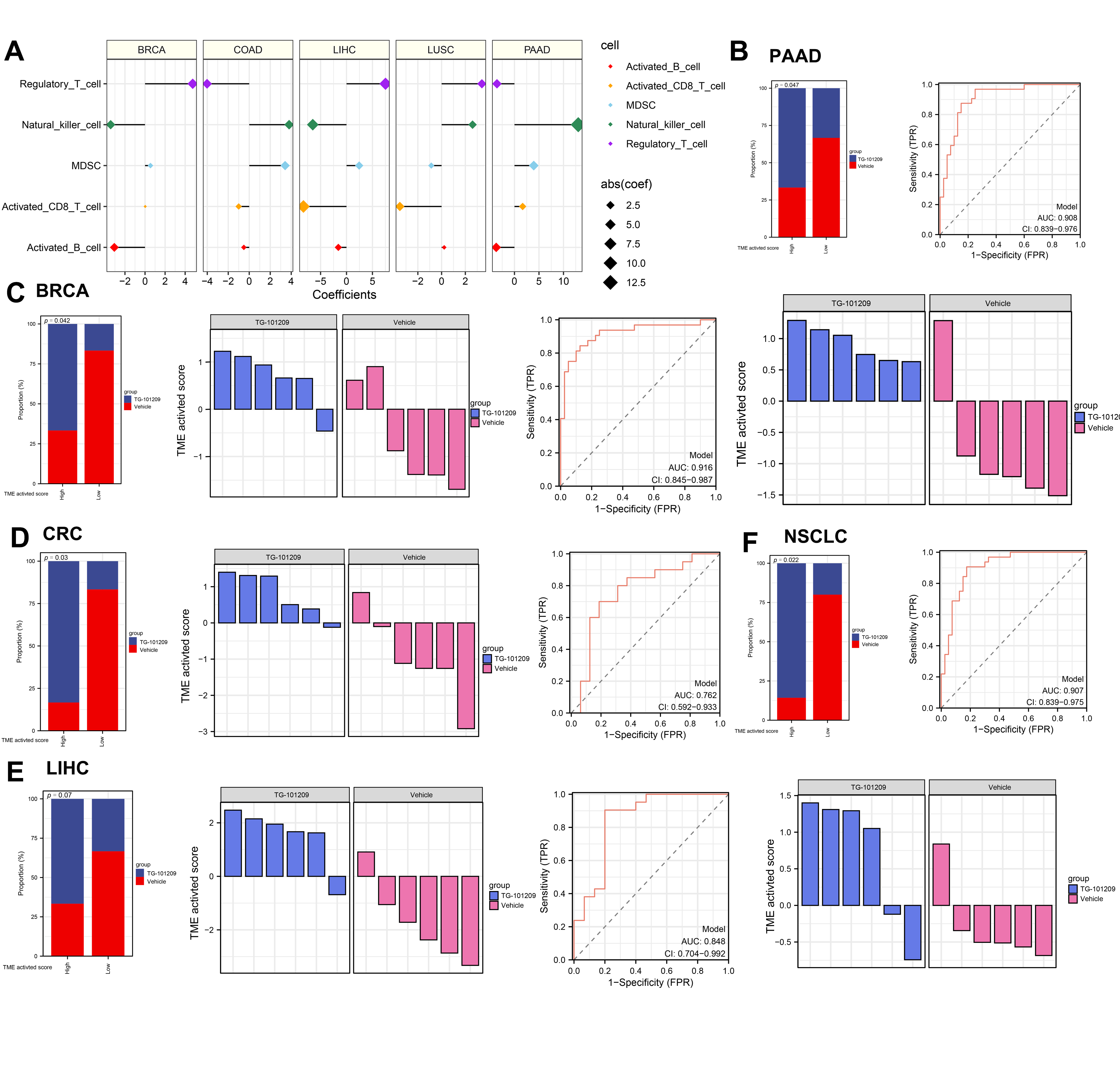

Supplement: Supplementary file 6 — Supplementary Material 6 [file 13046_2024_3111_MOESM6_ESM.tif]

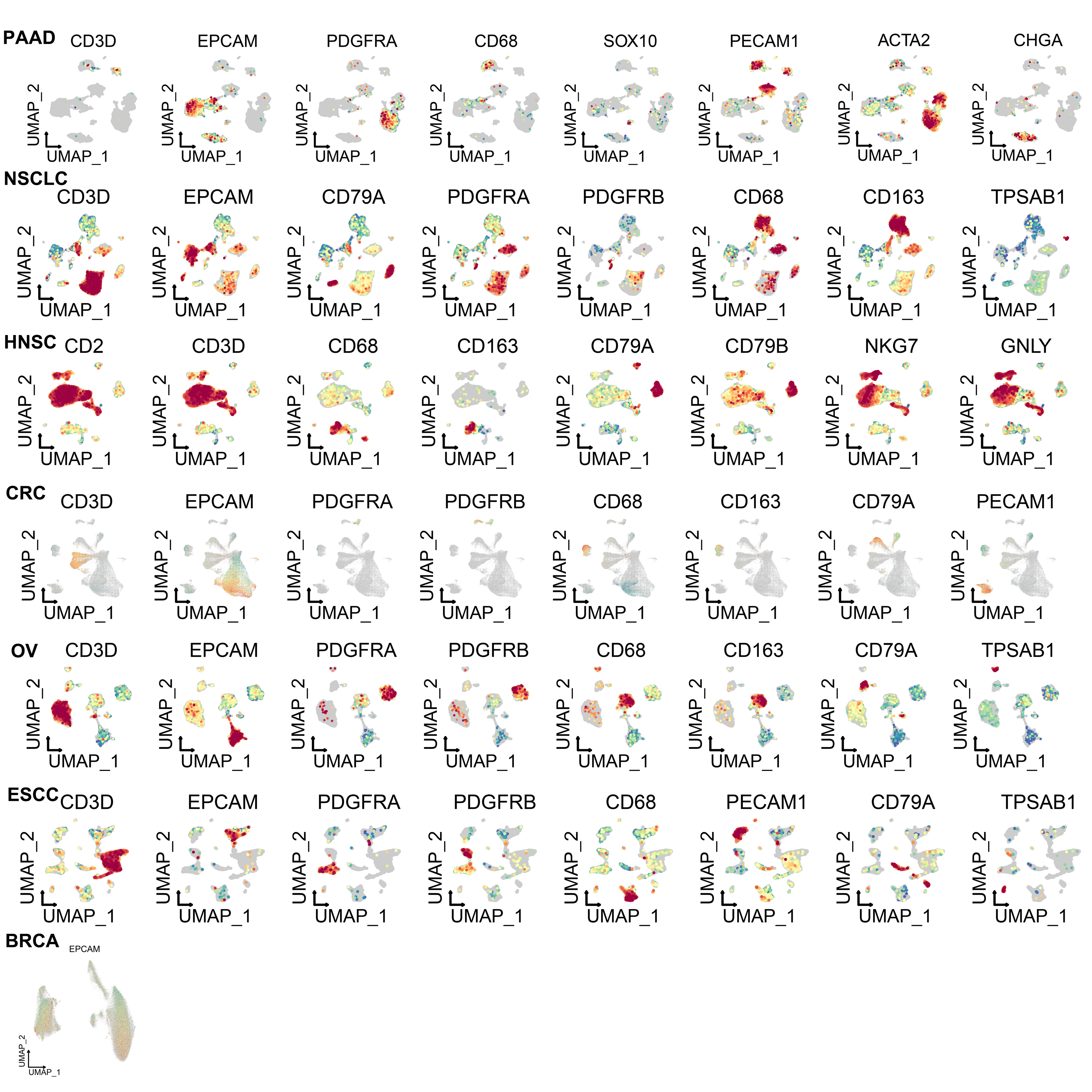

Supplement: Supplementary file 7 — Supplementary Material 7 [file 13046_2024_3111_MOESM7_ESM.tif]

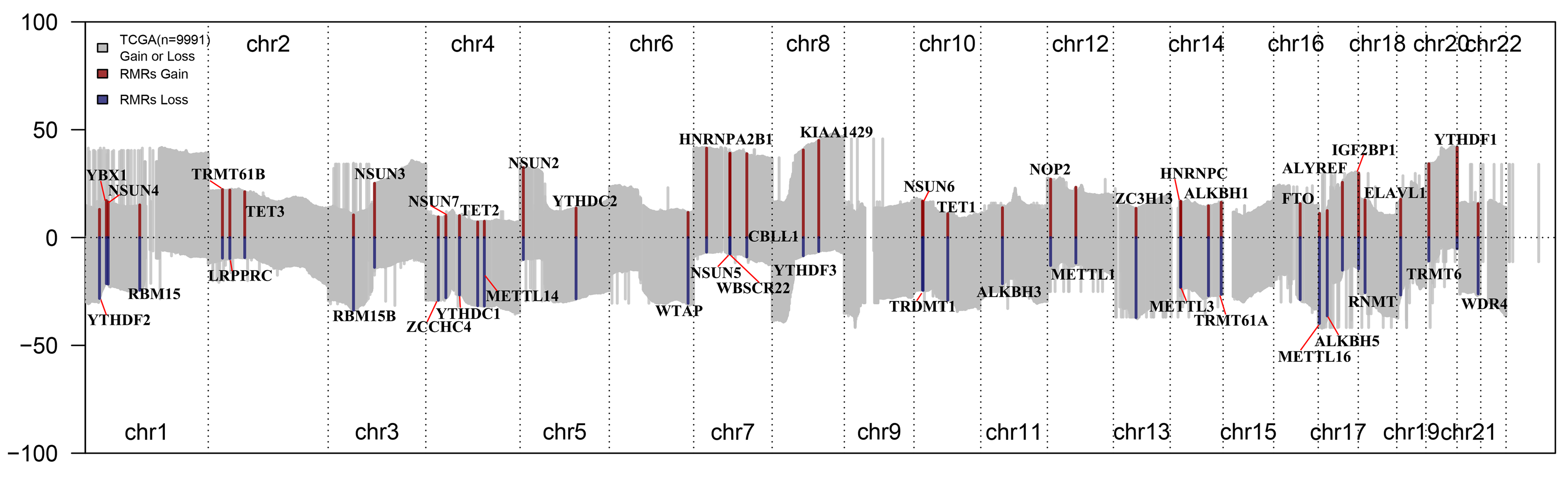

Supplement: Supplementary file 8 — Supplementary Material 8 [file 13046_2024_3111_MOESM8_ESM.tif]

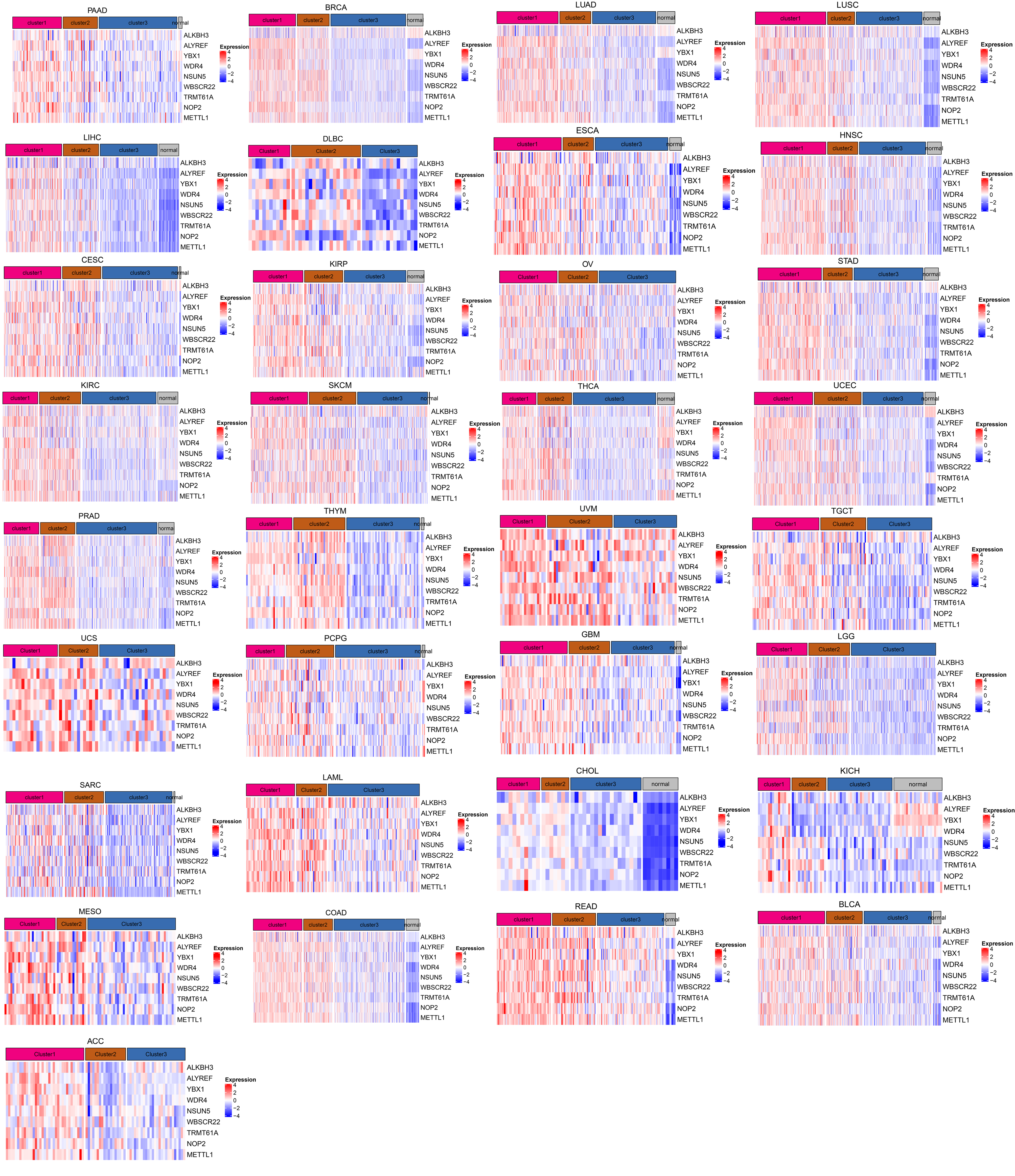

Supplement: Supplementary file 9 — Supplementary Material 9 [file 13046_2024_3111_MOESM9_ESM.tif]

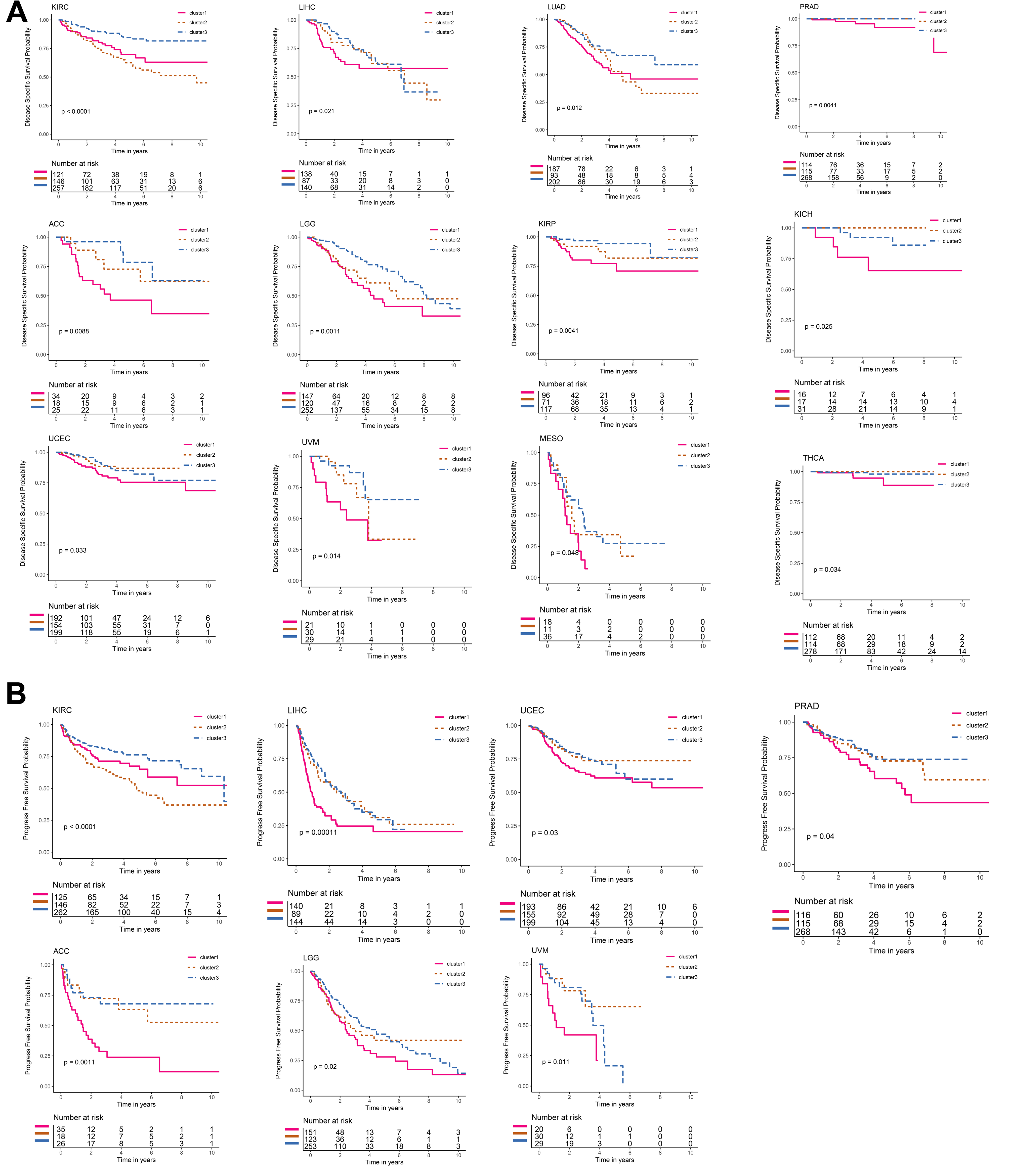

Supplement: Supplementary file 10 — Supplementary Material 10 [file 13046_2024_3111_MOESM10_ESM.tif]

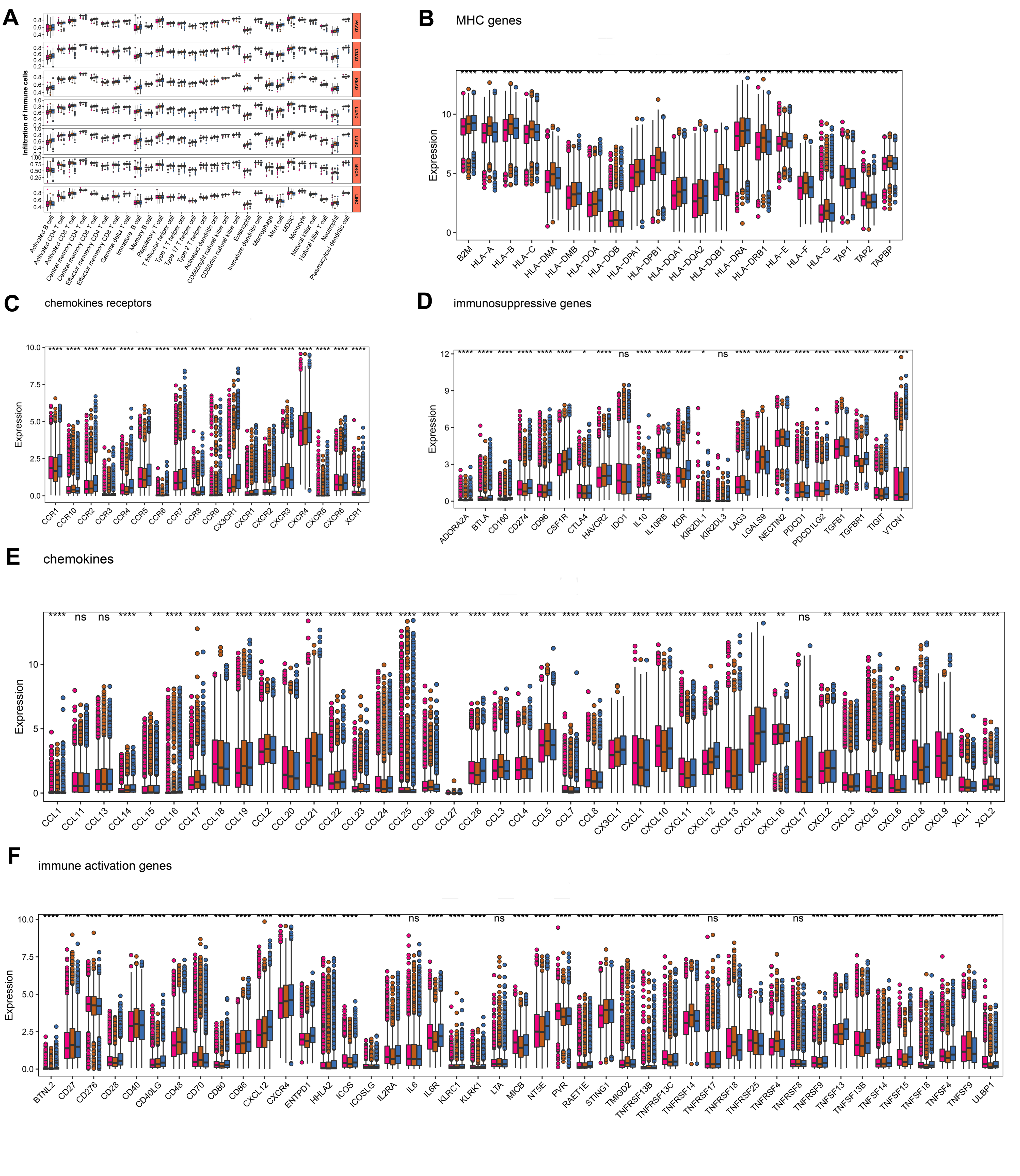

Supplement: Supplementary file 11 — Supplementary Material 11 [file 13046_2024_3111_MOESM11_ESM.tif]
