# Supplementary material for: Targeting treatment resistance: unveiling the potential of RNA methylation regulators and TG-101,209 in pan-cancer neoadjuvant therapy
Source: J Exp Clin Cancer Res. 2024 Aug 19;43:232. doi: 10.1186/s13046-024-03111-x (PMC11331809; doi:10.1186/s13046-024-03111-x)
Supplement: Supplementary file 19 — Supplementary Material 19 [file 13046_2024_3111_MOESM19_ESM.docx]

**Supplementary Figure legends**

**Fig.S1. Marker genes of cellular subpopulations in various cancer types, related to Fig.2.** UMAP plots showing representative gene expression levels in different cell subpopulations across seven cancer types.

**Fig.S2. CNV frequency of 46 RMRs in pan-cancer, related to Fig.3.** Density plot of the CNV frequency in 9991 TCGA samples. Red denotes the copy number gain while blue shows the copy number loss of 46 RMRs.

**Fig.S3. Expression profile of RMRs in RMRs clusters across cancer types, related to Fig.4.** Heatmaps showing expression levels of 9 RMRs in the Cluster1, Cluster2, Cluster3, and normal tissues in specific cancer types.

**Fig.S4. Disease-specific survival and Progression-free survival of RMRs clusters across cancer types, related to Fig.5. A-B.** Kaplan-Meier curves showing disease-specific survival and progression-free survival in different clusters across 12, and 7 cancer types respectively.

**Fig.S5. Immune gene expression in different clusters, related to Fig.6. A.** Boxplot of immune cells abundance in various cancer types by ssGSEA algorithm **B-F.** Box plots show different expressions of MHC genes**,** chemokines receptors**,** immunosuppressive genes, chemokines, and immune activation genes in three RMR clusters. P values between groups were determined by the Kruskal-Wallis test. NS P>0.05, *P<0.05, **P<0.01, ****P<0.0001.

**Fig.S6. Annotation of single-cell datasets and pathway enrichment, related to Fig.6. A.** Bubble plot showing typical gene expression of different cell types across five single-cell datasets. **B.** Bubble plot depicting GO and KEGG pathway enrichment.

**Fig.S7. Ability to compare RMRs and other features at the single-cell level to assess treatment response, related to Fig.7. A.** UMAP plot of different cell types in colorectal cancer dataset (GSE205506). **B.** UMAP diagram showing the different clusters of epithelial cells. **C.** Bar graph comparing the accuracy, AUC, recall, precision, and F1 score ( known as the Balanced Score, which is defined as the harmonic mean of precision and recall) of RMRs and other features in predicting the effect of chemotherapy response. **D.** Boxplots comparing the differences in scores for each gene set between the response and non-response. **E-F.** The UMAP plot demonstrates the fractional activity of RMRs in different epithelial subpopulations **(E)** of response and non-response **(F)**. **G.** Stacked bar graph comparing the difference in the percentage of microenvironmental cells between the two groups. **H.** Bubble and circle diagrams demonstrate the interactions of RMRs+/- epithelial cells with other cells. **I.** UMAP plot of different cell types in ovarian cancer dataset (GSE165897). **J.** UMAP plot showing different epithelial cell clusters. **K.** Comparative bar graphs of the performance of different gene sets in predicting treatment response in patients with ovarian cancer and box plots of high and low scores in different subgroups. **L.** Distribution of RMRs scores in different epithelial subpopulations. **M.** UMAP plot showing RMRs score in response and non-response. **N.** The bar plot displays the fraction of different cell types in response and non-response. **O.** The bubble plot of RMRs+/- epithelial cells interacting with other cell types. **P.** Circle plots show the strength of interactions between different cells and the interactions of MIF pathway signaling.

**Fig.S8. Ability to compare RMRs and other features at the cell line and bulk sample level to assess treatment response, related to Fig.7. A.** Heatmap showing AUC values for RMRs and other features predicting chemotherapeutic drug sensitivity in pan-cancer cell lines. **B.** Heatmap showing AUC values for RMRs and other features predicting chemotherapeutic drug response in samples from the pan-cancer TCGA dataset. **C-N.** ROC curves comparing the AUC values of RMRs and other genes for predicting the effects of chemotherapy **(C-G)** and immunotherapy **(H-N)**, with AUC representing the area under the ROC curve.

**Fig.S9. Pro-tumorigenic role of NOP2 in multiple tumors and its potential target drugs, related to Fig.7. A.** NOP2 expression in three RMRs clusters and normal tissues of different cancer types. **B.** The forest plots show an association of NOP2 with the prognosis of various cancer patients. **C.** GSEA analysis of NOP2 positively and negatively correlated pathways. **D.** CMap predicted small molecule drugs targeting NOP2.

**Fig.S10. TG-101209 affects RMRs’ transcriptome levels and protein levels of the JAK-STAT signaling pathway,** **related to Fig.9.** **A.** Western blots show the levels of proteins involved in the JAK-STAT signaling pathway in the TG-101209 treatment and control groups. P stands for phosphorylated. **B-J.** Bar graph demonstrating the differences in gene expression of RMRs between the TG-101209-treated and control groups with and without HJC0146 drug intervention. * P<0.05, ** P<0.01, *** P<0.001.

**Fig.S11. Relationship between TG-101209 treatment and tumor microenvironment activation, related to Fig.10. A.** Lollipop plot showing the coefficients of the calculated immune activation scores for five immune cells in each cancer type. **B-F.** Bar graph comparing the difference in the percentage of treatment and control groups in the high and low immune activation groups. Positive and negative bar graphs documenting TME-activated scores in each mouse model in the TG-101209 and control groups. ROC curves demonstrating TME-activated scores predicting AUC values for TG-101209 and controls.
